# Efficient design and analysis of secure CMOS logic through logic encryption

**DOI:** 10.1038/s41598-023-28007-2

**Published:** 2023-01-20

**Authors:** Sai Srinivas Chandra, R. Jagadeesh Kannan, B. Saravana Balaji, Sreehari Veeramachaneni, Sk. Noor Mahammad

**Affiliations:** 1 Department of CSE, Indian Institute of Information Technology Design and Manufacturing (IIITDM) Kancheepuram, Chennai, 600127 India; 2 School of Computer Science and Engineering, Vellore Institute of Technology Chennai Campus, Chennai, 600127 India; 3 Department of Information Technology, Lebanese French University, Erbil, 44001 Iraq; 4 Department of ECE, Gokaraju Rangaraju Institute of Engineering and Technology, Hyderabad, India

**Keywords:** Engineering, Electrical and electronic engineering

## Abstract

Untrusted third parties and untrustworthy foundries highlighted the significance of hardware security in the present-day world. Because of the globalization of integrated circuit (IC) design flow in the semiconductor industry, hardware security issues must be taken to prevent intellectual property (IP) piracy. Logic encryption is an efficient method to protect circuits from IP piracy, reverse engineering, and malicious tampering of IC for Trojan insertion. Researchers have proposed many logic encryption methods, which lead to overhead in circuit design parameters such as area, power, and performance. This paper aims to bring a trade-off between these parameters, with security being the main key factor, and ensure the design metrics by proposing a novel transistor-level method of logic encryption for CMOS gates. Experimental results show that, on the usage of proposed encrypted key gates, the design overheads such as area, power, delay, and energy are reduced by an average of 42.94%, 37.37%, 26.79%, and 50.96%, respectively, over the existing logic encryption-based topologies.

## Introduction

The main design requirement for an integrated circuit (IC) relies on methods that bring a trade-off between circuit performance and its compatibility. The primary concern in the present-day world is all about hardware trust^1^. The security of computer hardware, in particular, Integrated Circuits (IC), is an important aspect of the overall security of computer systems. Construction of a foundry with well-equipped and advanced fabrication capabilities requires much maintenance and involves high construction costs. As a result, fabless companies are sending their ICs to advanced and well-equipped foundries for fabrication^1^.

Consequently, an untrusted IC foundry may build ICs and sell them illegally. Further, once the chip enters the IC supply chain, it is also vulnerable to various reverse engineering attacks^2^, aiming to extract the design or specific secrets from a design like secret keys. Since the attackers are aware of the IC design flow, they can quickly reverse engineer the functionality of an IC/IP. Nowadays, hardware is prone to certain new kinds of attacks, including reverse engineering and IP piracy. Therefore, IP vendors face many challenges in protecting IPs from piracy, reverse engineering, and overproduction.

An untrusted foundry can do intellectual property (IP) theft, IC counterfeiting, IC overproduction, and also the insertion of hardware Trojans^3^ through malicious tampering of ICs^1,4,5^. As per statistics, the semiconductor industry loses 4 billion dollars annually because of all these problems^6^. Semiconductor Equipment and Materials International (SEMI) has done a survey recently, which states that almost 90% of the companies have experienced IP infringement, of which 54% of them report these issues as a severe and potential concern in terms of hardware security.

Rapid increment in the number of hardware-based attacks has brought up the need for hardware security to be considered and power, cost, performance, and reliability optimizations. Fabrication cost, power consumption, performance, and reliability must be considered while designing an IC. Hardware security emerged almost in 2007-2008 to protect IC/IP from threats in the semiconductor supply chain^7–9^. Since then, it has been gaining popularity among hardware security researchers^10,11^. A recent program on “Obfuscated Manufacturing of GPS” has been started by DARPA (Defense Advanced Research Projects Agency) to develop circuit locking methodologies against untrustworthy foundries^12^. We can encrypt the hardware functionality by a technique called logic encryption^4,10,13–15^ in which only authorized persons can access the original functionality, thus protecting the circuit from all forms of piracy. Logic encryption is an emerging area of research that cares about security norms in cases like IP theft, IC counterfeiting, IC overproduction, and hardware Trojans^7–9,16–19^. If an IC designer can hide an IC’s functionality when it passes through random untrustworthy phases of IC design flow, these attacks can be thwarted. The logic encryption technique involves the random insertion of key gates supplied with key bits such that the encrypted circuit will reveal the original functionality only for the correct key pattern.

This paper aims to implement a novel method of transistor-level encryption for CMOS logic style. On the application of an incorrect key pattern, an encrypted circuit will provide faulty outputs. Consequently, if an attacker somehow manages to obtain a circuit netlist by reverse engineering, he cannot obtain the original netlist until he gets the correct key. We propose a new structure for CMOS gates with two transistors for each gate with a little trade-off on circuit design considerations such as area, power, and delay alongside the security aspect. Contributions of this paper are mentioned as follows:

1. Detailed analysis of the work reported in the literature and implementing existing circuit topologies for CMOS.
2. Proposal of novel and secure circuit topologies for CMOS logic style aims to provide a decent trade-off between circuit parameter overheads and security inclusion.
3. We compared the proposed secure topology with existing gate-level encryptions such as XOR and LUT, and the area overhead is reduced from a minimum of 52.87% (as observed in the case of XOR) to a maximum of 74% (from LUT). Similarly, power consumption and circuit delay are reduced from 52.63 to 65.16% and 32 to 50.61%, respectively. Meanwhile, encryption standards for the circuit are not degraded.
4. Similarly, from the analysis, when comparing with proposed secure topology with transistor-level topologies such as Stack and Key-based encryption, the overheads area, power, and delay parameters are significantly reduced by 22.44%, 15.85%, and 12.28%, respectively.

This work’s main focus is to propose a novel circuit topology for implementation in CMOS logic style and demonstration of security features in terms of circuit functional and logical behavior. Researchers have proposed a few methodologies on logic encryption which typically uses XOR/XNOR gates^4,10,13,20,21^ as encryption, usage of AND/OR gates^22^, the addition of a look-up-table utilizing $4 \times 1$ MUX to a gate^14^, Stack-based topologies^23,24^ have also been proposed for few gates, and researchers have proposed key gate methodologies for CMOS logic against hardware Trojans^25^.

While all these methods have brought up some security concerns, few resulted in huge overhead and need to be more compatible in terms of security. Few resulted in poor security against logical circuit behavior in circuit topology. A novel transistor-level implementation is proposed in this paper, which reduces circuit overhead compared with existing models without being compromised on circuit security aspects of logical, structural, and functional behavior.

The structure of the paper begins with an introduction to hardware security and logic encryption. Current logic encryption methodologies at the gate and transistor level are provided in “Gate level logic encryption” section. An overview of design considerations and the overheads of described logic encryption techniques are presented in “Analysis of literature work” section. An explanation of the proposed topology is provided in “Proposed logic encryption topology” section. A comparative discussion of results between the proposed topology and the existing topologies is described in “Results and discussion” section. Finally, conclusions and future work are given in “Conclusion” section.

Logic encryption methodologies exists in both combinational and sequential circuits^7–9,16–19,26,27^. Combinational logic encryption focuses on key insertion in the targeted circuit to encrypt the original functionality. Unless the key is correct, one cannot obtain the original functionality. Also, combinational logic encryption involves changes in the logic within the circuit, whereas sequential logic encryption involves applying a sequence before the circuit’s correct operation is obtained.

### Gate level logic encryption

Many researchers have proposed a few design methodologies for combinational logic encryption at the gate level. There are two important gate-level encryption methodologies proposed to encrypt circuit functionality.

1. Usage of LUT in the form of MUX for Encryption.
2. Encryption using XOR/XNOR gates.

**Figure 1:**
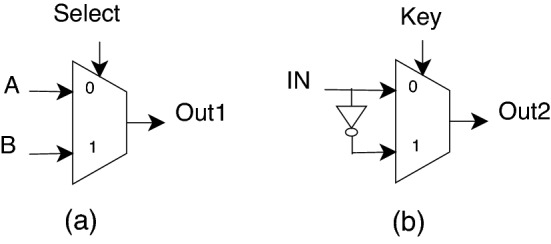
2:1 MUX based encryption.

Additionally, researchers have a proposed 2:1 MUX-based logic encryption^13,15,28^ also, in which correct output is connected to one input of MUX and the other input of MUX gets the inverted version of the output. The selection input acts as a key input. The multiplexer’s operation is to propagate one of the input signals to the output based on the input selection. Figure 1a shows the circuit diagram of the multiplexer. Figure 1b shows the encrypted multiplexer.

The key input, defined as “Key,” acts as a select line with two values, either 0 or 1, which gives IN or negated IN at output “Out2”. The truth table for this 2:1 MUX-based Encryption is shown in Tables 1, 2.

**Table 1:** Truth table of encrypted 2:1 MUX.

Input	Input	Output
Key	IN	OUT2
0	0	0
0	1	1
1	0	1
1	1	0

**Table 2:** Truth table for XOR encrypted circuit.

Inputs		Key	Output	Key	Output
A	B	K	Out	K	Out
0	0	0	0	1	1
0	1	0	0	1	1
1	0	0	0	1	1
1	1	0	1	1	0

Finding a net that is always the inversion of the correct input is challenging, limiting the application of 2:1 MUX-based logic encryption.

#### LUT based logic encryption

**Figure 2:**
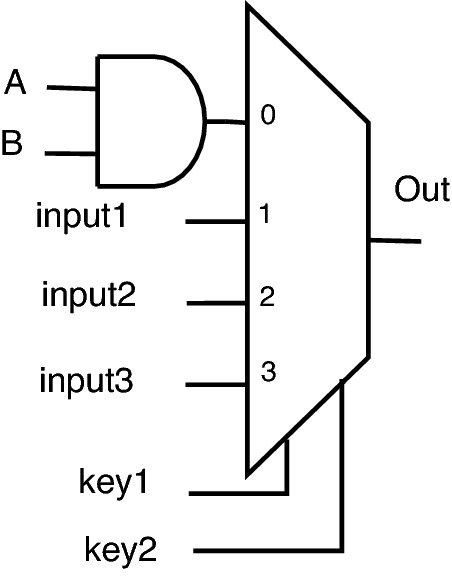
LUT encryption.

Researchers have proposed a methodology that uses a 4:1 MUX^14^ to encrypt the circuit functionality in the form of a look-up-table structure. This method involves the circuit (CMOS gate here) followed by a LUT, as shown in Fig. 2.

**Figure 3:**
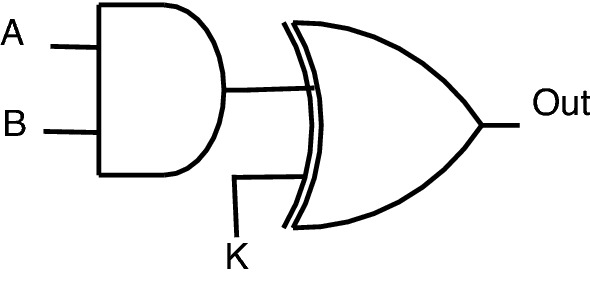
XOR encryption.

Here in this structure, there will be a usage of two key inputs, key 1 and key 2, as select lines of the MUX, and out of 4 input lines of MUX, one of the input lines gets the gate output as shown in the Fig. 2. The input lines here stated as “input1, input2, input3” can have either 1 or 0 depending on the circuit designer. For simplification purposes, we have assumed the MUX inputs as 1, 0, and 1, respectively. MUX’s select lines, which act as key inputs, will have four possible key combinations: 00, 01, 10, and 11. This method adds security to get the correct functionality unless we have the two correct keys, key 1 and key 2, as mentioned. Here, in this case, the correct keys are 00 to get AND gate as output. The output function is incorrect for the rest of the three other values of keys, such as 01, 10, and 11. The encrypted circuit logical behavior is given as a truth table from Table 3.

**Table 3:** Truth table of encrypted LUT.

Gate inputs		Key inputs		MUX inputs			MUX output
A	B	Key1	Key2	Input1	Input2	Input3	Out
0	0	0	0	1	0	1	0
0	1	0	0	1	0	1	0
1	0	0	0	1	0	1	0
1	1	0	0	1	0	1	1
0	0	0	1	1	0	1	1
0	1	0	1	1	0	1	1
1	0	0	1	1	0	1	1
1	1	0	1	1	0	1	1
0	0	1	0	1	0	1	0
0	1	1	0	1	0	1	0
1	0	1	0	1	0	1	0
1	1	1	0	1	0	1	0
0	0	1	1	1	0	1	1
0	1	1	1	1	0	1	1
1	0	1	1	1	0	1	1
1	1	1	1	1	0	1	1

The replication of corresponding input lines of MUX to “Out” is observed on applying incorrect keys. One undesirable major problem with this encryption is the considerable circuit design overheads due to increased transistor count.

#### XOR based logic encryption

XOR-based encryption methodology^4,10,13,20,21^ includes a structure of a circuit (CMOS gate here) followed by an XOR or XNOR gate. The output of the circuit goes to one of the two inputs of the XOR, the other input being a key input “K,” which encrypts the circuit functionality as illustrated in Fig. 3. The logical circuit behavior can be analyzed through the truth table, as shown in Table 2.

This encrypted circuit behavior is depicted as follows:

1. When key input K is − 1, the circuit functionality will not get disturbed.
2. When key input K is 0, the circuit’s inverted output will be obtained at “Out.”

In another way, the XOR gate behaves as an inverter when the key input is 1, and the XOR gate acts as a buffer when the key input is 0. This encryption methodology also generates massive overhead due to increased propagation path circuitry.

### Transistor level encryption

The motivation for implementing transistor-level encryption is because of the huge overheads faced in gate-level encryption. If there is a possibility of inserting key gates at the transistor level, then there can be reduced overhead in terms of design considerations. Insertion of extra gates in the logical path like XOR-based encryption^4,10,13,20,21^, or inserting a massive circuitry like LUT-based encryption^14^ adds a large area overhead. Also, it results in additional levels of logic, which may reduce the performance of the circuitry.

Researchers have proposed two principal methodologies to encrypt the circuit at the transistor level by adding key gates inside the circuit^23–25^. As a result, the correct key gives the original functionality, and faulty key results in incorrect circuit behavior.

To the best of our knowledge, there are two such methods proposed:

1. Stack Based Logic Encryption.
2. Key Based Logic Encryption.

#### Stack based logic encryption

**Figure 4:**
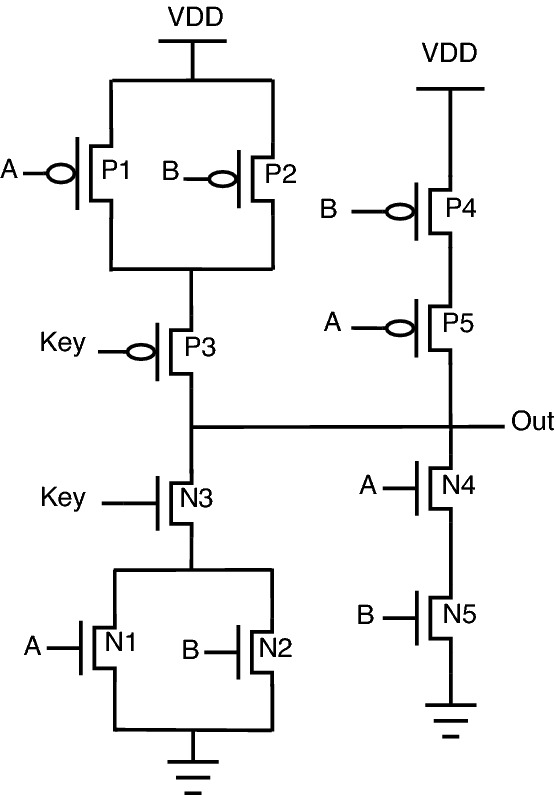
Stack NAND-NOR.

The stack-based topology^23,24^ is depicted for the NAND-NOR gate, as shown in Fig. 4. In this topology, the key gate’s value decides which stack (either PMOS stack or NMOS stack) to be activated. PMOS stack consists of P1, P2, and P3 transistors; similarly, the NMOS stack consists of N1, N2, and N3 transistors. The circuit behavior is described as follows:

1. When “Key” is 0, the PMOS stack is activated, allowing the gate to behave as NAND.
2. When “Key” is 1, the NMOS stack is activated, allowing the gate to behave as NOR.

**Figure 5:**
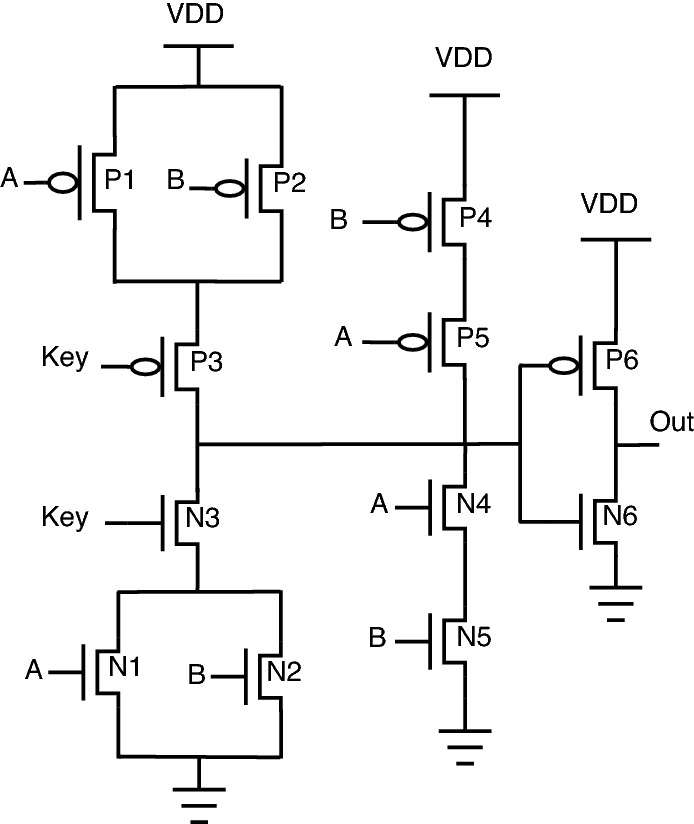
Stack AND-OR.

One of the reasons for placing the “Key” transistors on the “Out” net of the gate is that it reduces the capacitance connected to “Out” by essentially disconnecting one of the logic stacks during execution. A smaller output capacitance reduces performance and power overhead through this stack-based encryption topology. On the same lines, AND-OR stack-based topology can be framed using an inverter, as shown in Fig. 5.

This topology’s primary benefit is that only one of the stacks gets disconnected from “Out”, which limits the capacitance connected to “Out”. This results in a reduction of power consumption and also limits degradation in performance. Another benefit of this type of stack configuration is that there will be a shared functionality between the gates’ implemented logic. The circuit functionality for stack-based encrypted circuits can be understood from the truth Table 4.

**Table 4:** Truth table for stack based encryption.

Inputs		Key input	Output	
A	B	Key	NAND-NOR Out	AND-OR Out
0	0	0	1	0
0	1	0	1	0
1	0	0	1	0
1	1	0	0	1
0	0	1	1	0
0	1	1	0	1
1	0	1	0	1
1	1	1	0	1

For example, the NAND and NOR gates have the same logical output when inputs A and B are both 0 or 1, which permits shared functionality as indicated by the line of transistors P4, P5, N4, N5 (shared functionality) shown in Fig. 4.

The shared functionality is obtained from P4, P5, N4, and N5 transistors. There is no requirement for a key transistor in the shared functionality as the NAND and NOR produce the same output for the 00 and 11 input combinations. Using the stack-based approach, the ability to go without using key transistors reduces the circuit parameter overheads. One more important property of this stack-based topology is that it does not require negated inputs, removing two more transistors and reducing overhead. Those negated inputs are required if NAND/AND topology is implemented. In this case, the negated logic is needed as the same input combinations must turn on a PMOS or NMOS stack, depending on the key.

A similar topology for AND/OR is also implemented by adding an inverter at the output, as shown in Fig. 5. While both the characteristics of stack-based topology contribute to reducing design overheads, sharing the two common output cases when inputs A and B are both 0 or 1 reduces the percentage of inputs that produce incorrect outputs when an incorrect key is applied.

#### Key gate based logic encryption

**Figure 6:**
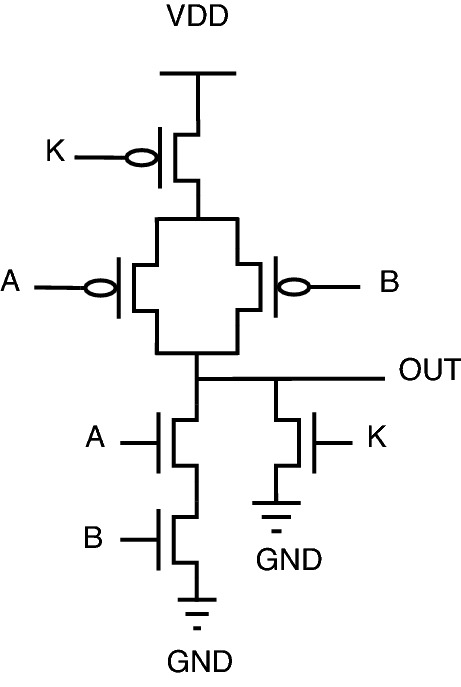
NAND gate.

**Figure 7:**
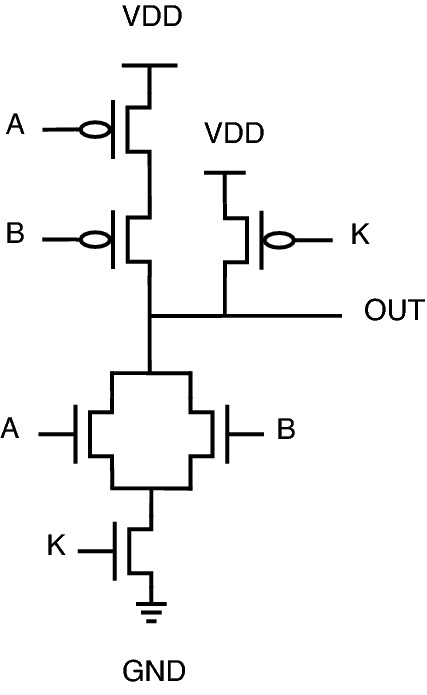
NOR gate.

**Figure 8:**
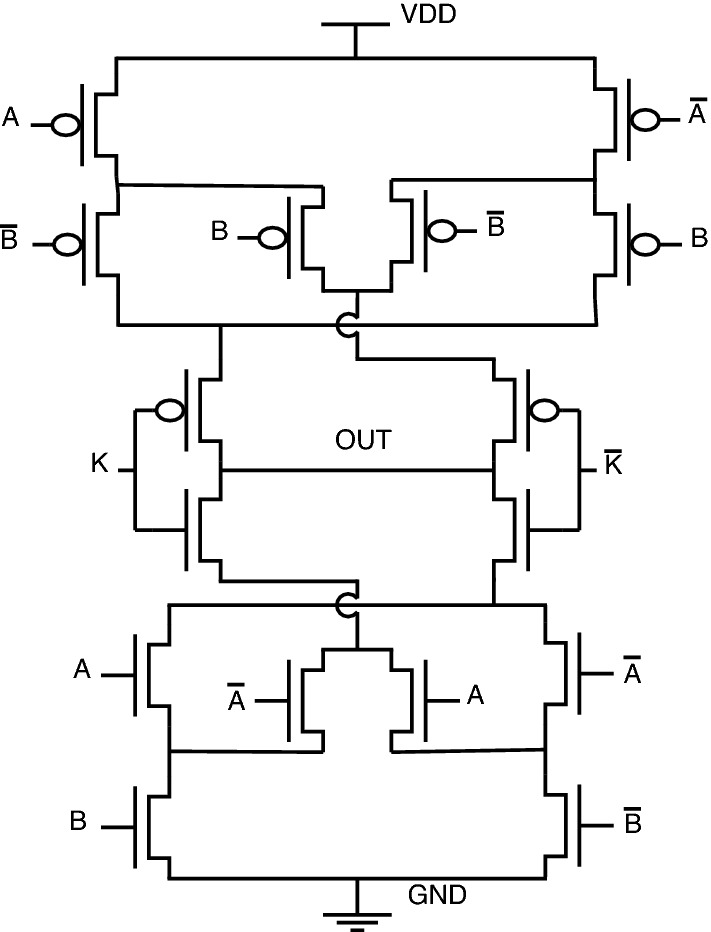
XOR ST gate.

**Figure 9:**
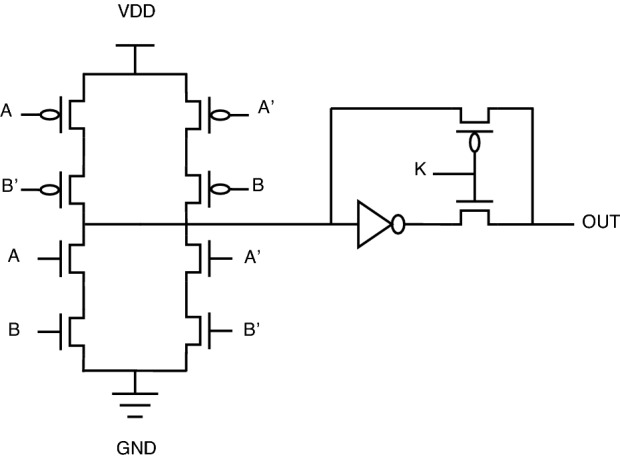
XOR PT gate.

Researchers have also focused on the efficient way of preventing hardware Trojans^25^, which resulted in a new topology at the transistor level. This topology also uses key gates inside the circuit to encrypt the original functionality. The correct key will give the original functionality. The correct key may be either 0 or 1. Schematic diagrams of the key-based logic gates have been illustrated below from Figs. 6, 7, 8, 9.

**Table 5:** Truth table for key based encryption.

Inputs		Key	Outputs				
A	B	K	AND	NAND	OR	NOR	XOR/XNOR
0	0	0	1	1	0	1	0
0	1	0	1	1	1	1	1
1	0	0	1	1	1	1	1
1	1	0	1	0	1	1	0
0	0	1	0	0	0	1	1
0	1	1	0	0	0	0	0
1	0	1	0	0	0	0	0
1	1	1	1	0	0	0	1

Here, in this case, the valid key for AND/NOR is 1, whereas the valid key for OR/NAND is 0.

In this encryption, two topologies have been designed for XOR (XOR PT and XOR ST) with valid key 0. XOR gate with pass transistor topology (XOR PT) and stack-based topology (XOR ST) provides a trade-off between area and logic value. Similarly, XOR gate topology can also be used for XNOR by changing inputs.

As observed from truth Table 5, the application of an invalid key provides a constant “1” for AND/NOR gates, and similarly invalid key for OR/NAND provides a constant “0”. When it comes to the case of XOR/XNOR, inversion of the correct functionality is obtained for the application of an incorrect key. Conventional OR/NAND will have a low probability of output being “0”, but when comparing conventional OR/NAND circuitry with this key-based encrypted circuitry, the key-gate provides a constant “0” to increase the value of output probability being “0”. Similarly, when compared with conventional AND/NOR, key-based encrypted gates will increase the output probability of “1” by providing a constant “1”. This circuit behavior can be observed from Table 5. The major drawback of this topology is that the key is a partial part of the circuit.

## Analysis of literature work

The discussion of overheads in design considerations such as area, power, and delay in the cases of XOR, LUT, Stack, and Key-based encryption topologies is described in this section. For comparison and analysis purposes, design considerations of unencrypted standard cell CMOS gates are also included along with literature and proposed work. The circuits presented in this paper are implemented using a 45nm technology library in Cadence Virtuoso 6.1 Tool.

The following simulation characteristics were applied to all the circuits presented in the paper:

- All simulations were completed with a load capacitance of 1fF.
- The area which is obtained is from the layouts of the implemented topologies.
- The propagation delay is considered the worst-case delay obtained from the transient analysis of the implemented topologies.
- The average power is determined from the power analysis of the corresponding topologies.
- The implemented circuits are analyzed using a 45nm technology node, ensuring standard transistor width and length.

Please note that there is no use of memory elements for LUT based encryption approach. The values provided for each method’s circuit design metrics are highly optimistic compared to the implementations that use memory elements.

The per-gate overheads for all CMOS gates in terms of area, power, and delay for all the mentioned encryption styles are analyzed and listed in Tables 11, 12, 13, 14, 15, 16, 17, 18, 19, 20, 21, 22. The huge overheads indicated in Tables 11, 12, 13, 14, 15, 16, 17, 18, 19, 20, 21, 22 resulted in limiting XOR, LUT-based encryption models in IC applications.

The XOR-based approach adds an extra XOR gate to the unencrypted gate, where data must go through an extra stage to get the output, further increasing the area and reducing performance. The LUT-based approach replaces the XOR gate with a 4*1 MUX. The XOR-based approach has 1 key input, whereas the LUT-based approach has 2 key inputs. Even though security is enhanced, poor performance and design metrics resulted in a limitation of LUT based approach. Also, this approach involves additional transistors required for the implementation. As a manufactured IC is required to meet the industrial design standards of circuit compatibility and reliability constraints, large overheads are less desirable even after considering security standards of logic encryption. Therefore, bringing down the overheads and maintaining the security norms as per logic encryption methodology is essential.

Researchers propose two unique transistor-level topologies, stack-based, and key gate-based logics, which significantly reduce overheads and increase performance compared to XOR and LUT-based approaches. Stack-based topology is of good use when all the input combinations do not require the generation of incorrect output when an incorrect key is applied. If there is a requirement of incorrect output for the incorrect key, stack-based topology is not beneficial as it results in tremendous power and area overhead. Key-based topology focuses on the security and design metrics, resulting in a new topology for logic encryption methodologies. This topology gives better performance when compared with gate-level XOR and LUT-based encryption methodologies. One major problem with this topology is that continuous logic high (1) or low (0) is observed at the output when an incorrect key is applied to a few gates, which is undesirable. This topology provides immunity against the trojan attack with minimal design overheads compared with existing topologies. However, it may need to improve at preventing piracy, overbuilding, and reverse engineering.

**Figure 10:**
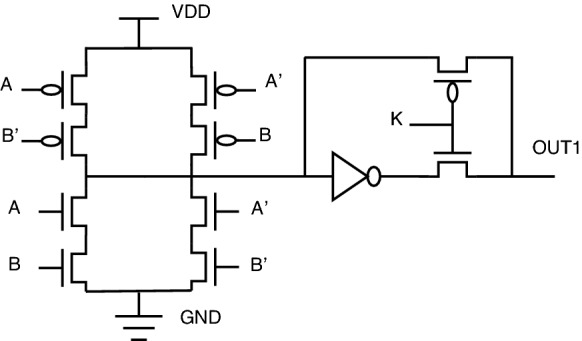
XOR PT: without buffer.

**Figure 11:**
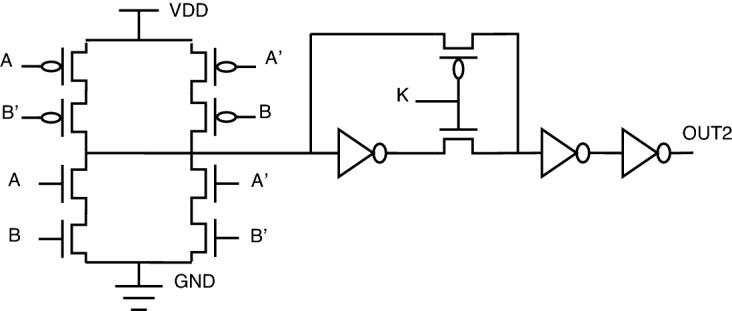
XOR PT: with buffer.

One particular issue with key-based transistor-level topology is a voltage-level degradation problem associated with the so-called XOR PT topology. Because of the pass transistor topology implemented at the output of this XOR PT circuit, the circuit’s logical behavior is not proper. Instead, there will be a lot of signal distortion. As per our investigations, this topology needs a buffer circuit to restore voltage signals to full swing. The scenario explained can be illustrated in Figs. 10 and 11.

A similar problem exists for XNOR also. The same solution of adding a buffer circuit will help in solving the voltage signal degradation problem in the existing encrypted gate topology.

## Proposed logic encryption topology

This section presents the proposed designs with efficient architecture for CMOS gates at the transistor level, followed by the implementation of gate designs with their structural and functional analysis.

**Figure 12:**
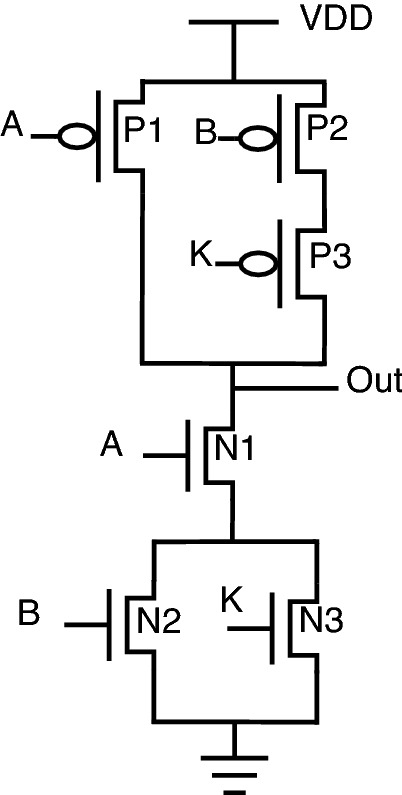
Secure NAND.

**Figure 13:**
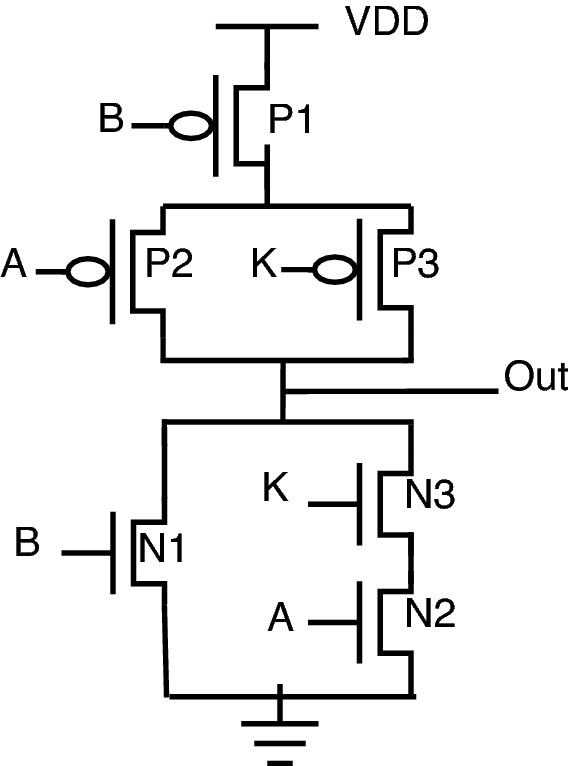
Secure NOR.

**Figure 14:**
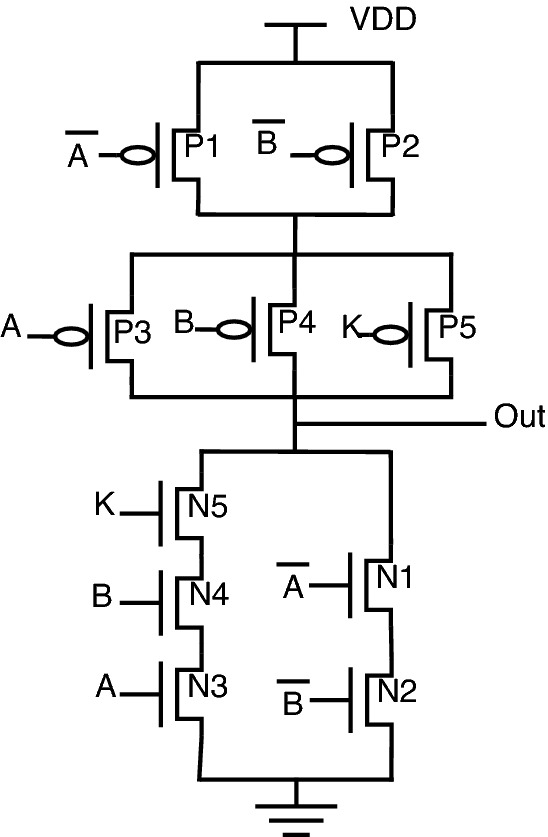
Secure XOR.

**Figure 15:**
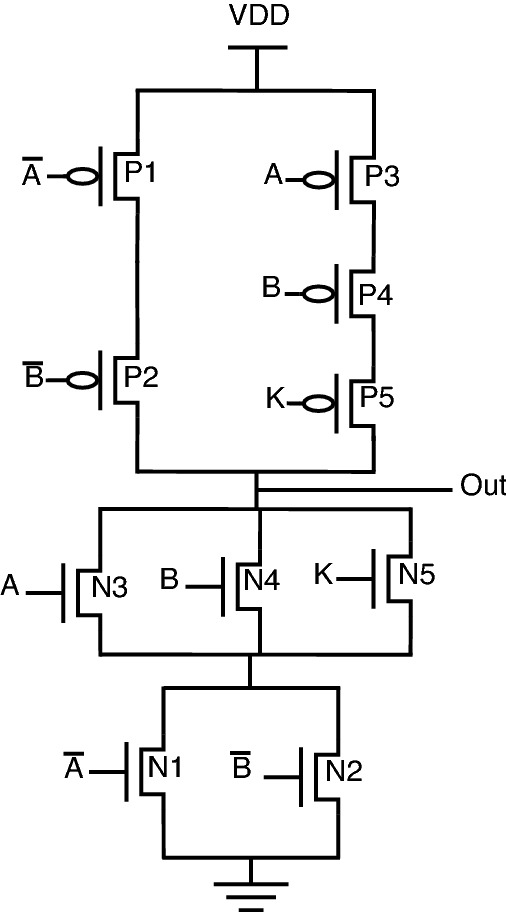
Secure XNOR.

**Figure 16:**
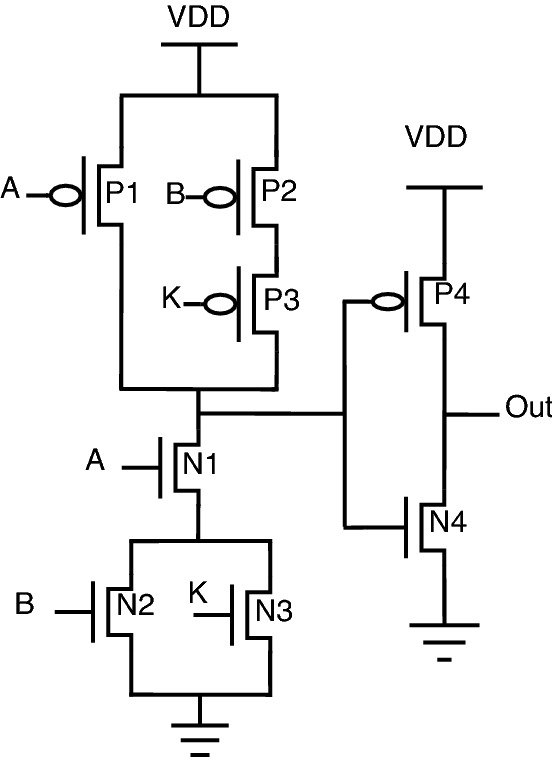
Secure AND.

**Figure 17:**
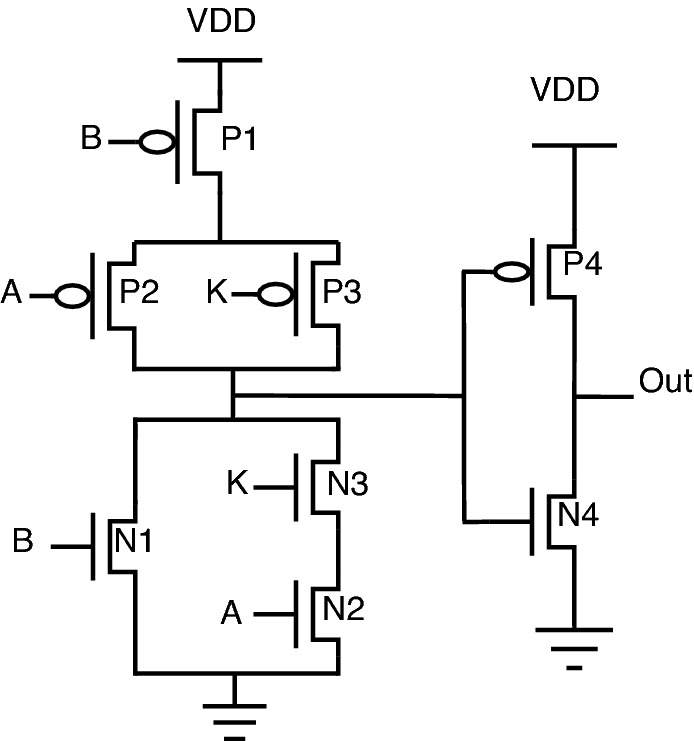
Secure OR.

The schematic diagrams of the proposed encrypted gate topologies for NAND, NOR, XOR, XNOR, AND, and OR gates are shown from Figs. 12, 13, 14, 15, 16, 17, respectively. Gates’s functional behavior can be observed from schematics and the truth table for both correct and incorrect keys, which brings off a decent security feature making it difficult for an attacker to decrypt the design through layout, netlist, or by reverse engineering. It can be observed that the circuit gives correct functionality only when a valid key is applied and incorrect behavior for the invalid key.

For encryption of logic gates, there is a minimum requirement of two key gates to be included in the circuit to achieve security. The significant advantage distinguishing this novel design from the existing methodologies is that this proposed topology requires only two key gates for encryption. The proposed encrypted circuit topologies achieve this minimum requirement of including two key transistors to achieve secure circuit functional behavior. The functionality of the proposed circuits with the valid and invalid key is listed in Table 6. The valid key for OR, NOR, and XOR is “1”. For AND, NAND, and XNOR, the valid key is “0”. As it is observed from the schematics of proposed secure CMOS circuit designs, there is only a minor change in structure, which is nothing but the addition of two key transistors, N3, P3 for AND, OR, NAND, NOR, and N5, P5 for XOR, XNOR topologies.

This addition of two key transistors will have less overhead in the circuit area, power consumption, and performance. Consider the proposed NAND gate for circuit analysis, as shown in Fig. 12. As we go through circuit functional behavior, we have P1, P2, and P3 transistors in the pull-up network, with A, B, and K being the gate inputs, respectively. Similarly, N1, N2, and N3 are in a pull-down network.

The circuit functional behavior has 2 cases (K = 0 and K = 1), which can be analyzed as follows:

- Case-1: When key input K is “0”, the circuit behaves as NAND without any deviation in logical behavior.
- Case-2: When key input K is “1”, the original NAND functional behavior is encrypted and provides faulty circuit behavior.

In short, we can say that for applying an incorrect key, output “Out” will be obtained either as an inversion of one of the inputs “A or B” or the inputs themselves. The only minor structural change is the inclusion of two key transistors, one in a pull-up network and the other in a pull-down network, to balance the concept of CMOS logic, thus achieving proper secure circuit functional and logical behavior.

Consider proposed NOR gate, as shown in Fig. 13, the circuit functional behavior can be analyzed as follows:

- Case-1: When K is “1”, the circuit works as NOR gate.
- Case-2: When key input K is “0”, the original NOR behavior is masked with one circuit input.

One unique behavior is observed from the proposed encrypted XOR and XNOR gates. One advantage of these proposed circuits is the inclusion of two-gate functionality into a single circuit.

The circuit behavior for the proposed XOR is as follows:

1. When K is “0”, the circuit will function as an OR gate.
2. When K is “1”, the circuit will function as an XOR gate.

Similarly, if we observe the proposed XNOR gate functionality:

1. When K is “0”, the circuit will function as an XNOR gate bringing up the original circuit functionality.
2. When K is “1”, the circuit will function as AND gate, thereby masking out the actual circuit behavior.

The similar approach can be used to look into circuit functionality for AND and OR gates. The proposed circuits shown in Figs. 16 and 17 utilizes inverter topology to implement gate functionality. To avoid inverters at the output, proposed XOR and XNOR gates serve the functionality of OR and AND, respectively, upon incorrect keys.

The functional circuit analysis and logical behavior can be observed from truth Table 6.

As can be observed from the previous literature on encrypted circuits, the key is a partial part of the circuit. For the mentioned literature circuits, the attacker can easily find the key as the key is not built. However, here in this proposed topology, the key is built as a complete part of the circuit, and the circuit function will be disrupted by removing the key input. This property of key addition in the circuit’s internal structure can be stated as one of the advantages of proposed circuits.

**Table 6:** Truth table of proposed gates.

Key	Inputs		Outputs					
K	A	B	AND	OR	XOR	NAND	NOR	XNOR
0	0	0	0	0	0	1	1	1
0	0	1	0	1	1	1	0	0
0	1	0	0	0	1	1	1	0
0	1	1	1	1	1	0	0	1
1	0	0	0	0	0	1	1	0
1	0	1	0	1	1	1	0	0
1	1	0	1	1	1	0	0	0
1	1	1	1	1	0	0	0	1

, One other advantage of this topology is re-arranging inputs, which further results in a change of output. Consider the proposed NAND gate for analysis purposes. Since there are 3 inputs, A, B, and K, there is a possibility of re-arranging these 3 inputs in 3 different patterns, which can be observed from Figs. 18, 19, and 20. The following cases can be stated by re-arranging inputs.

- *Case-1:* Considering standard proposed circuit without any re-arrangement of circuit inputs.
- *Case-2:* When circuit inputs are re-arranged in a pattern different from case-1.
- *Case-3:* When circuit inputs are re-arranged into the third pattern different from above cases 1 and 2.

*Case-1: Considering standard proposed circuit model* Consider the proposed NAND gate, as shown in Fig. 18, for analysis, assuming that this is the standard model for comparison. Considering the pull-up network, circuit inputs are arranged as A-P1, B-P2, and K-P3. Similar patterns can be observed for the pull-down network as well.

**Figure 18:**
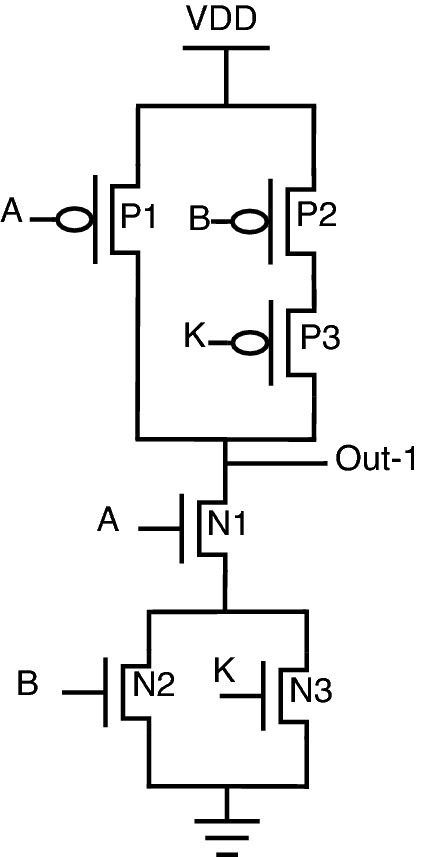
Case-1.

**Figure 19:**
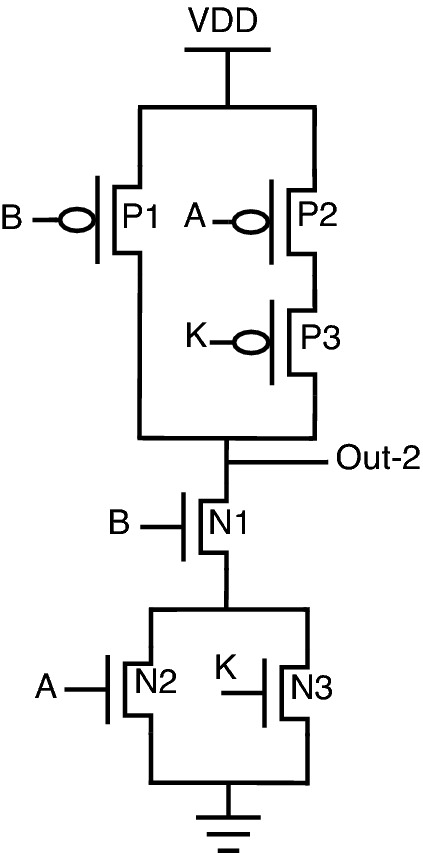
Case-2.

**Figure 20:**
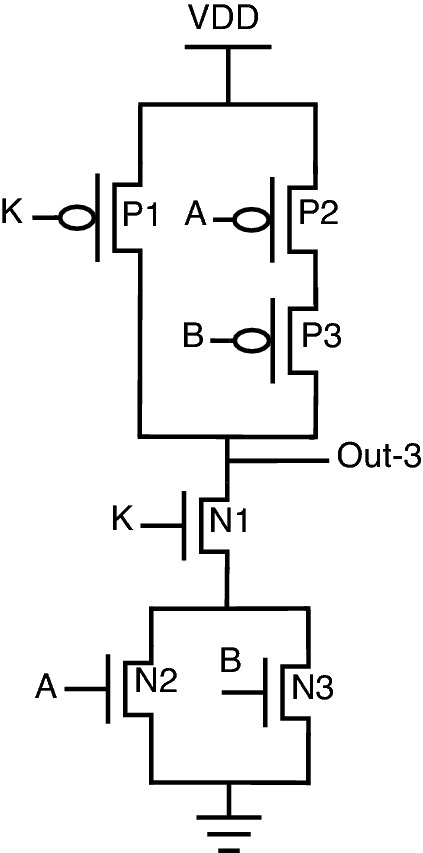
Case-3.

As observed from truth-table 7 for case-1, the circuit function as conventional NAND for key = 0, and for key = 1, which is an incorrect key, the circuit will provide an output pattern of 1,1,0,0 for corresponding input combinations.

*Case-2: When circuit inputs are re-arranged in a pattern different from case-1* Regarding case-2, as depicted in Fig. 19, circuit inputs are re-arranged in the order of B-P1, A-P2, and K-P3 for the pull-up network, and a similar pattern can be observed for pull-down network as well. For this model, key = 0 gives correct functionality, and for key = 1, the circuit provides a pattern of 1,0,1,0. For the application of incorrect key in cases 1 and 2, the output pattern obtained is different.

This logical behavior can be observed as “Out-1” and “Out-2” from truth-table 7.

*Case-3: When circuit inputs are re-arranged into the third pattern different from above cases 1 and 2* This model comes with an input pattern of K-P1, A-P2, and B-P3 in a pull-up network with the same pull-down pattern observed in Fig. 20.

This case is a special one, which provides other gate functionality on re-arranging inputs. In short, the circuit will function as NOR when key = 1, and for key being 0, the circuit will provide a constant “1,” thereby increasing the probability of output is “1”.

The logical circuit behavior can be observed from truth-table 7 under “Out-3”. There is a complete difference in outputs just by interchanging inputs of the proposed circuit. This property of interchanging inputs will create a dilemma for an attacker to trace the circuit functionality.

**Table 7:** Truth table for analysis of proposed NAND gate.

Key	Inputs		Outputs		
K	A	B	Out-1	Out-2	Out-3
0	0	0	1	1	1
0	0	1	1	1	1
0	1	0	1	1	1
0	1	1	0	0	1
1	0	0	1	1	1
1	0	1	1	0	0
1	1	0	0	1	0
1	1	1	0	0	0

Similarly, if we consider all possible interchanging input cases for all proposed circuits, we obtain the logical behavior, as shown in Table 8. One more significant advantage of special gates XOR and XNOR is multi-functionality. Through interchanging inputs, XOR can function as XOR, OR, and NAND. Similarly, XNOR can function as XNOR, AND, and NOR. This functional behavior can be observed from truth Table 8. This property can be stated as one of the advantages of the proposed topology, which is not observed from literature circuits.

**Table 8:** Truth-table of proposed gates.

Inputs			Outputs																	
K	A	B	NAND			NOR			AND			OR			XOR			XNOR		
			C1	C2	C3	C1	C2	C3	C1	C2	C3	C1	C2	C3	C1	C2	C3	C1	C2	C3
0	0	0	1	1	1	1	1	1	0	0	0	0	0	0	0	0	0	1	1	1
0	0	1	1	1	1	0	1	1	0	0	0	1	0	0	1	1	0	0	0	0
0	1	0	1	1	1	1	0	1	0	0	0	0	1	0	1	0	1	0	0	0
0	1	1	0	0	1	0	0	0	1	1	0	1	1	1	1	1	1	1	0	0
1	0	0	1	1	1	1	1	0	0	0	0	0	0	1	0	1	1	0	0	0
1	0	1	1	0	0	0	0	0	0	1	1	1	1	1	1	1	1	0	1	0
1	1	0	0	1	0	0	0	0	1	0	1	1	1	1	1	1	1	0	0	1
1	1	1	0	0	0	0	0	0	1	1	1	1	1	1	0	0	0	1	1	1

### Circuit analysis using proposed key gates

This section presents the efficiency of proposed key gates by incorporating them into the circuit and analyzing their logical behavior at various output stages. This circuit-level analysis using proposed key gates demonstrates how well incorrect outputs mask the original outputs at different levels. This circuit analysis can be explained in two possible ways, as mentioned:

1. Circuit analysis demonstrating the key efficiency.
2. Circuit analysis through output probability.

#### Circuit analysis demonstrating the key efficiency

The proposed gates’ efficacy in masking out the output at various circuit levels can be understood when the proposed key gates are incorporated into a circuit, replacing standard gates. For analysis, consider the following two cases:

- Circuit analysis with standard CMOS gates.
- Circuit analysis with proposed CMOS gates.

**Figure 21:**
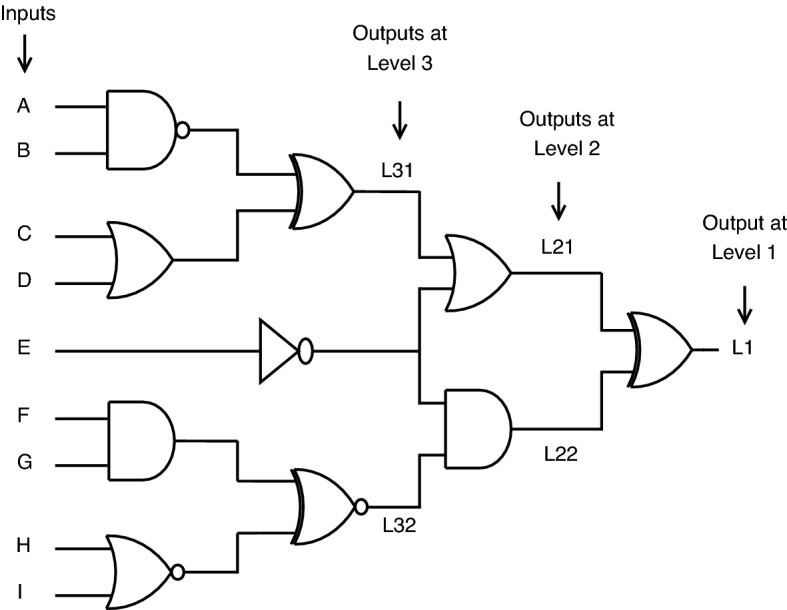
circuit with standard CMOS gates.

*Circuit analysis with standard CMOS gates* Consider the combinational circuit shown in Fig. 21 for which A, B, C, D, E, F, G, H, and I are taken as inputs. L1 is considered as output obtained at level 1. L21 and L22 are the outputs obtained at nodes of level 2. Similarly, L31 and L32 are outputs at level 3, respectively.

For circuit analysis in terms of logical behavior, a total of 10 test cases are considered. The output obtained at various levels (such as L1, L21, L31) of the circuit can be analyzed from the truth Table 9.

**Table 9:** Truth table for standard CMOS circuit analysis.

Test	Inputs									Outputs				
Case	A	B	C	D	E	F	G	H	I	L31	L32	L21	L22	L1
1	1	0	1	1	0	1	1	0	1	0	0	1	0	1
2	0	1	1	0	1	0	1	1	0	0	1	0	0	0
3	1	1	0	1	0	1	0	1	1	1	1	1	1	0
4	0	0	0	0	1	1	1	0	0	1	1	1	0	1
5	1	1	0	0	0	1	1	0	0	0	1	1	1	0
6	0	0	1	1	1	0	0	1	1	0	1	0	0	0
7	1	0	0	1	0	0	1	1	0	0	1	1	1	0
8	0	1	1	0	1	1	0	0	1	0	1	0	0	0
9	1	1	0	0	1	1	0	0	1	0	1	0	0	0
10	1	1	0	0	0	1	1	0	1	0	0	1	0	1

**Figure 22:**
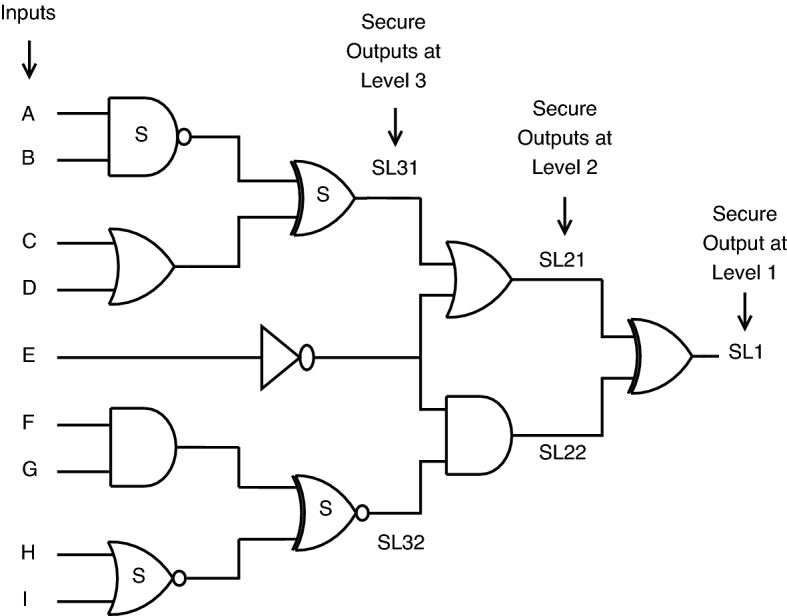
circuit with proposed CMOS gates.

*Circuit analysis with proposed CMOS gates* Consider the circuit shown in Fig. 21, which brings a minor change from Fig. 21. This circuit in Fig. 22 involves replacing a few standard CMOS gates with proposed secure gates, which are highlighted with the letter “S,” stating them as secure. The inputs are the same for both circuits. Here, in this case, SL0 stands for secure output at level 1. SL21 and SL22 are secure outputs obtained at level 2. Similarly, SL31 and SL32 are for level 3.

There are two possible cases for proposed gates with the correct or incorrect key. For the correct key, secure gates exhibit logical behavior, as shown in Table 8. For incorrect key, the secure gates’ logical behavior masks out the original output with faulty one at different levels (such as SL1, SL21, SL31) of output, as seen from the truth Table 10.

From the truth Tables 9 and 10, many differences are noticed from the following result pairs L31-SL31, L32-SL32, L21-SL21, L22-SL22 and L1-SL1.

The test cases are the same for both circuits. A lot of output variation is observed upon applying proposed key gates, which states that the proposed key gates successfully mask the original functionality of a circuit with faulty outputs, thereby building secure circuits that prevent access to attackers.

**Table 10:** Truth table for secure CMOS circuit analysis.

Test	Inputs									Outputs				
Case	A	B	C	D	E	F	G	H	I	SL31	SL32	SL21	SL22	SL1
1	1	0	1	1	0	1	1	0	1	1	0	1	0	1
2	0	1	1	0	1	0	1	1	0	1	0	1	0	1
3	1	1	0	1	0	1	0	1	1	1	0	1	0	1
4	0	0	0	0	1	1	1	0	0	1	1	1	0	1
5	1	1	0	0	0	1	1	0	0	0	1	1	1	0
6	0	0	1	1	1	0	0	1	1	1	0	1	0	1
7	1	0	0	1	0	0	1	1	0	1	0	1	0	1
8	0	1	1	0	1	1	0	0	1	1	0	1	0	1
9	1	1	0	0	1	1	0	0	1	0	0	0	0	0
10	1	1	0	0	0	1	1	0	1	0	0	1	0	1

Our analysis observed that this encrypted circuit confuses the attacker to decrypt the circuit to obtain original functionality. The attacker cannot obtain the original circuit netlist because of the proposed gates’ unique output patterns. Also, a unique case of multi-gate functionality for special gates such as the proposed XOR and XNOR.

#### Circuit analysis through output probability

One other way through which the efficiency of proposed gates can be analyzed is by calculating the output probabilities at each node of a circuit, thereby analyzing the circuit behavior. This can be done by incorporating proposed key gates by replacing the standard cell gates in a combinational circuit. For analysis, consider the following cases:

- Output probability analysis using standard gates.
- Output probability analysis using key-based gates
- Output probability analysis using stack-based gates
- Output probability analysis using proposed gates.

**Figure 23:**
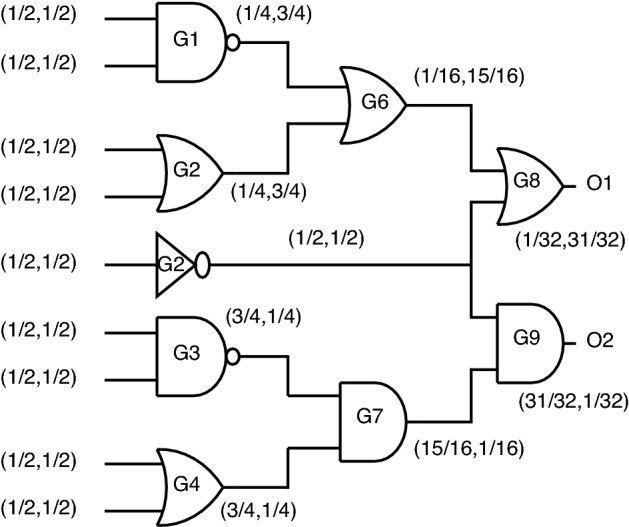
Output probability analysis using standard gates.

*Output probability analysis using standard CMOS gates* Circuit analysis in terms of output probability at each node using unencrypted standard cell CMOS gates in a gate-level combinational circuit is shown in Fig. 23.

Let (P0, P1) be defined as the probability of obtaining 0 and 1, respectively. As observed from Fig. 23, O1 and O2 are defined as output nodes for the entire circuit for which (P0, P1) are obtained as (1/32, 31/32) and (31/32, 1/32), respectively. This output probability obtained by standard gates helps the attacker easily to figure out which gate is present at the output node.

**Figure 24:**
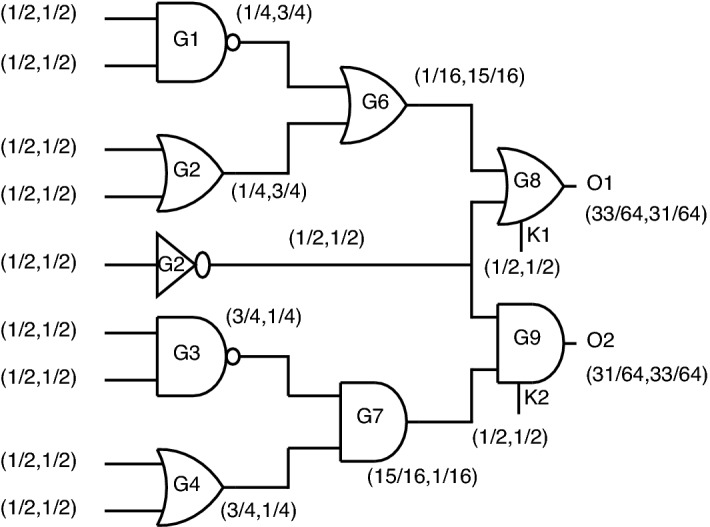
Output probability analysis using key-based gates.

*Output probability analysis using key based gates* When replacing standards gates at the output with key-based gates, the output probabilities are almost equalized, as shown in Fig. 24.

For the outputs, O1 and O2, the probabilities obtained in this case are (33/64, 31/64) and (31/64, 33/64), which creates a dilemma for attackers to figure out which gate is present at the output node. Even though these key gates function correctly at the gate level, this topology’s disadvantage is lacks security as individual gates, which produce constant “0” or “1” at output upon incorrect key application.

**Figure 25:**
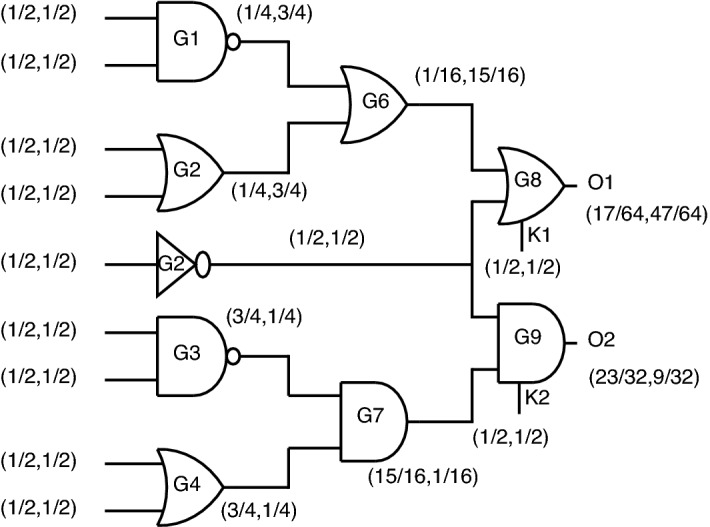
Output probability analysis using stack gates.

*Output probability analysis using stack-based gates* Stack-based gates provide an irregular pattern of probability when stack gates are incorporated into a complex circuit. As observed in Fig. 25, the output node probabilities were obtained as O1 (17/64, 47/64) and O2 (23/32, 9/32).

Stack gates have the advantage of two-gate functionality but fail in intricate circuit design as they could be more efficient in masking out the probability at circuit outputs. The Key is a partial part of the circuit, which is also a drawback for this topology. Stack topology is provided only for a few gates, such as NAND-NOR and AND-OR, failing to produce XOR and XNOR functionality, which can be stated as another drawback for stack encryption.

*Output probability analysis using proposed gates* The property of involving a key gate as part of the encrypted circuit makes this proposed topology effective in securing the circuit. The key being in-built into an encrypted circuit can be understood by interchanging inputs and observing the circuit behavior. When analyzing circuit behavior through output probability, the out probability also varies from node to node by interchanging inputs of the encrypted circuit. This scenario can be depicted as three cases, possibly stated in section 4, by considering the re-arrangement of inputs for the proposed NAND gate as an example.

- Case-1: Proposed standard circuit topology.
- Case-2: Re-arrangement of inputs from case-1.
- Case-3: Re-arrangement of inputs in a pattern differing from cases 1 and 2.

**Figure 26:**
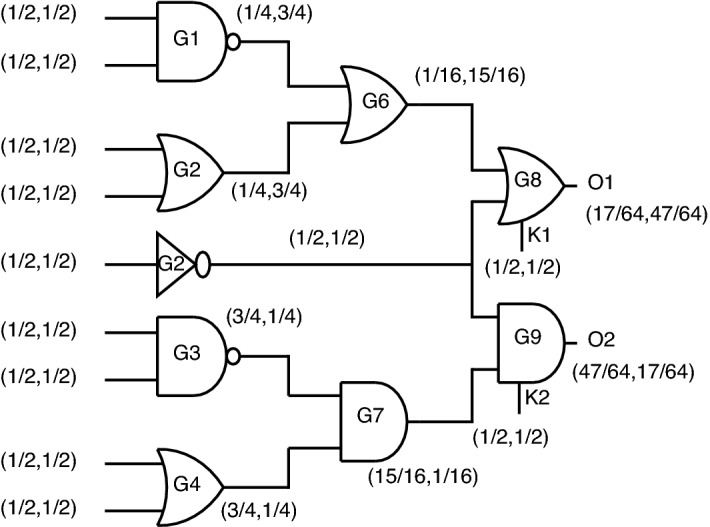
Case-1: Probability analysis using proposed gates.

*Case-1:* When placing proposed gates at the output of the combinational circuit, the probabilities at output nodes are obtained, as shown in Fig. 26.

For the outputs O1 and O2, the probabilities obtained in this case are (17/64, 47/64) and (47/64, 17/64). This is one of three possible cases of probability obtained at the output node when considering proposed gates.

**Figure 27:**
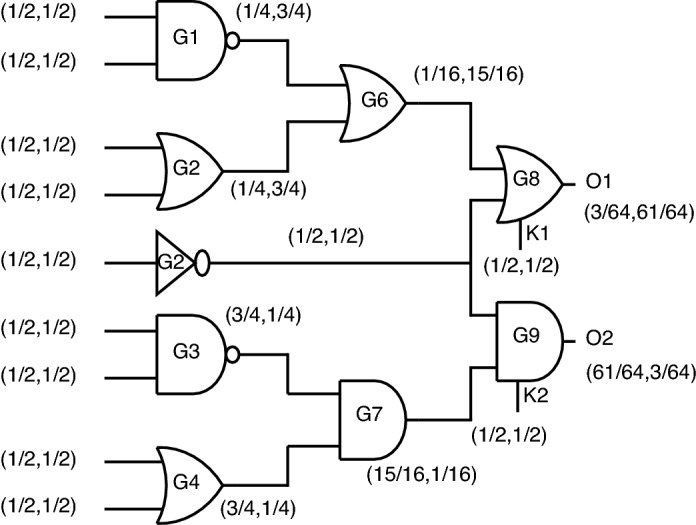
Case-2: Probability analysis using proposed gates.

*Case-2:* When considering the second case, the probability analysis is depicted in Fig. 27.

For the outputs O1 and O2, the probabilities obtained are (3/64, 61/64) and (61/64, 3/64). The second case is masking out the output probability from case-1 just by re-arranging inputs different from case-1.

**Figure 28:**
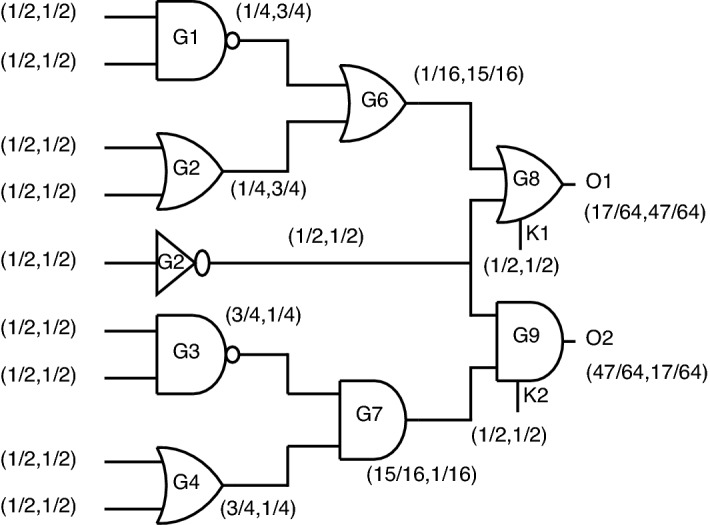
Case-3: Probability analysis using proposed gates.

*Case-3:* When considering the third case, the probability analysis can be seen from Fig. 28.

For the outputs O1 and O2, the probabilities obtained are (17/64, 47/64) and (47/64, 17/64).

Even though there is a match in probability from case-1 to case-3, the output function will change entirely. In this case, O1, which means the proposed OR gate, will produce AND functionality for the correct key. Similarly, O2, which is nothing but proposed, AND gate will function as OR gate. This special case brings a complete change in functionality without a change in output probability.

Finally, the proposed key gates are significant enough to mask out the output probability, circuit structure, and functional behavior, thus making them secure in every possible way. This theoretical analysis proves that the proposed circuits are resilient against SAT attacks.

## Results and discussion

The existing and proposed circuits presented in this paper are modeled and implemented using a 45nm technology library in Cadence Virtuoso 6.1 Tool. The per-gate overheads are listed in the table for the proposed design, which significantly shows a better design than existing design methodologies. The following are the prominent points concluding that the proposed design is a promising approach for logic-based transistor-level encryption:

- The major advantage of the proposed design is that an attacker will not insert a trojan into the netlist’s internal node because the attacker cannot access the original netlist.
- The proposed topology achieves a minimum requirement of two key transistors for bringing the security feature, which will not increase much overhead.
- The added advantage of this proposed topology is that the key is in-built, making it hard for the attacker to decrypt the circuit.
- Even by interchanging the key input with gate inputs, the proposed topology can function appropriately along with security.
- Similarly, if the proposed gates’ inputs are re-arranged in any manner, circuit functionality will change accordingly. This is because of the reason that the key is a whole part of the circuit structure.
- Logical behavior (as observed from truth Tables 6 and 8) of every proposed gate is different from each other, thereby creating a dilemma for an attacker to figure out the original gate functionality.
- The replacement of existing gate encryption topologies with the proposed topology will significantly prevent hardware trojan insertion.
- When compared to XOR, LUT-based logic encryption approaches, the proposed design method significantly reduces circuit overheads.
- Unlike stack-based topology, the proposed topology provides security for every gate by encrypting the original circuit functionality.
- The proposed topology serves as an efficient approach in logic encryption to prevent piracy, overbuilding, and reverse engineering.
- The output probability analysis of the proposed key gates stands as an efficient way to prevent attackers from decrypting the encrypted circuit.
- The circuit analysis using proposed gates at various output nodes provides a clear view of circuit obfuscation.
- Proposed key-based topology will not have any constant or continuous output, such as logic high (1) or logic low (0), as observed in the case of existing key-based topology.
- Inclusion of the XNOR gate’s multi-gate functionality behaving as both AND gate and XNOR gate with the key input difference. Similarly, the XOR gate behaves as XOR when the key input is 1, and when the key input is 0, it behaves like an OR gate.
- The proposed key gates provide security in various aspects, such as hiding circuit functionality, changing design structure, masking output probability, the inclusion of multi-gate functionality, and security at various circuit levels.
- Finally, the proposed key gates reduce energy (power delay product) consumption over all the mentioned design topologies.

To the best of our knowledge, there are mainly the following categories of comparison for CMOS logic encryption, considering literature work with our proposed encryption topology.

1. Standard CMOS Cell.
2. Gate Level LUT Topology.
3. Gate Level XOR Topology.
4. Transistor Level Stack Topology.
5. Transistor Level Keyed Topology.
6. Proposed Novel Topology.

The proposed novel transistor-level secure CMOS topology deals with adding 2 key transistors and provides a good amount of security in terms of logical circuit behavior. The proposed topology brings a good trade-off within design parameters compared with existing literature topologies, thereby making circuits more secure and less overhead in design considerations.

Comparison of circuit design metrics such as area, power, delay, and energy (Power-Delay Product) for each gate are listed in Tables 11, 12, 13, 14, 15, 16. A comparison of design overheads in terms of percentage changes for each gate is listed in Tables 17, 18, 19, 20, 21, 22.

For analysis, consider the following notations as mentioned:

- For area overhead analysis, “I” stands for an increment in the area when using the corresponding literature encryption methodology over the proposed encryption topology, and “R” stands for a reduction in the area when using standard unencrypted gates over the proposed encryption topology.
- For power overhead analysis, “I” stands for increment in power consumption when using the corresponding literature encryption methodology over proposed encryption topology, and “R” stands for a power reduction when using standard unencrypted gates over proposed encryption topology.
- For performance analysis, “I” stands for increased delay/reduced circuit performance when using the corresponding literature encryption methodology over the proposed encryption topology, and “R” stands for reduced delay when using standard unencrypted gates over the proposed encryption topology.
- Similarly, for energy (PDP as mentioned) consumption analysis, “I” stands for a percentage increment in overall energy consumption when using existing topologies over the proposed topologies. Similarly, “R” denotes a reduction in energy consumption when using standard unencrypted gates over the proposed encryption topology.

### Analysis of circuit design parameters

Circuit design parameters are essential in analyzing circuit compactability regarding industry standards. The CMOS results for all the encryption methodologies and proposed encryption are depicted in the following tabulations from Tables 11, 12, 13, 14, 15, 16. These tabulations contain circuit parameter-related information such as metric values for the area, power, delay, and PDP for standard unencrypted gates, literature encryption methodologies (key-based, XOR, LUT, and stack-based topologies), and also for proposed gates for comparison purposes.

There is only an unavoidable minor increment in parameters when compared to standard unencrypted cells. However, compared with existing literature on circuit topologies, there is a good trade-off in the proposed topology parameters. A significant reduction in parameters has been observed. Transistor count also stands as a metric for the area when analyzed as per industrial standards. The proposed topology of circuits results in lower energy consumption. Researchers have proposed stack-based topology only for limited gates such as NAND-NOR. For the same reason, we have considered stack topologies for NAND, NOR, AND, and OR gates only.

For XOR gate analysis from Table 15, two sub-topologies for the XOR gate are considered under key-based encryption methodology. One topology is a key-based XOR gate, as shown in Fig. 10. Another topology is made by considering the buffer circuit, as shown in Fig. 11. These topologies are stated as XOR (without buffer) and XOR (with buffer) in the Table 15. These two topologies are considered for analysis because they depict the drawbacks of the literature key-based circuits over the proposed circuits. A similar analysis for the XNOR gate is considered under key-based circuits, as shown in Table 16. Since there is a dual-gate functionality for proposed XOR and XNOR, XOR/OR notation is given in Table 15 and on a similar basis, XNOR/AND notation is given in Table 16 under the category of proposed encryption methodology. As can be observed from tabulations 11, 12, 13, 14, transistor count for key-based and proposed gates are the same, but the security property is enhanced a lot when considering proposed gates over the key-based gates.

### Analysis of percentage improvements

Analysis of percentage improvements provides information regarding the efficiency the proposed topology gains over the existing encryption topologies in terms of industrial circuit design metrics such as reliability, compatibility, and optimization. This analysis results can be observed from Tables 17, 18, 19, 20, 21, 22. These tabulations give an overview of percentage changes in circuit parameters when comparing the proposed topology with the standard unencrypted topology and literature circuit topologies.

Consider percentage analysis for AND gate as shown in Table 17, there are two comparison cases for analyzing percentage improvements.

- *Case-1*: Comparison of standard CMOS unencrypted gates with proposed encrypted gates.
- *Case-2*: Comparison of literature encrypted circuits with proposed encrypted gates.

Considering case-1, since there is an addition of 2 key transistors for every proposed gate over the standard CMOS gates, there is a minor reduction in circuit parameters that account for corresponding percentage changes when comparing proposed gates over the standard CMOS gates. As observed from Table 17, there is a change of 25% area reduction, 1.92% reduction in power consumption, and 4.44% performance improvement observed for standard unencrypted CMOS gates when compared with the proposed gate topology. These parameter changes are unavoidable since security is a major concern now.

Now considering case-2, which involves literature circuit analysis, there is a change of 55.14% area overhead, 49.92% of power consumption, 31.81% performance degradation, and 65.86% of energy consumption is observed when using XOR-based AND gate over the proposed AND gate. These overheads are indicated in tabulations 17, 18, 19, 20, 21, 22 with the letter “I” indicating the increment in overheads over the proposed circuit design. Similarly, there is a change of 76.2% area overhead, 65.83% of power consumption, 55% performance degradation, and 84.62% of energy consumption observed when using the LUT-based AND gate over the proposed AND gate. A similar analysis can be used to analyze key- and stack-based AND over the proposed AND gate. The percentage change in area is “0” for key-based CMOS because key-based CMOS AND gate and proposed AND gate utilize the same number of transistors, accounting for 0 percentage change in the area when comparing the topologies. The transistor count, which accounts for both the AND gates’ circuit area, can be observed from 11.

**Figure 29:**
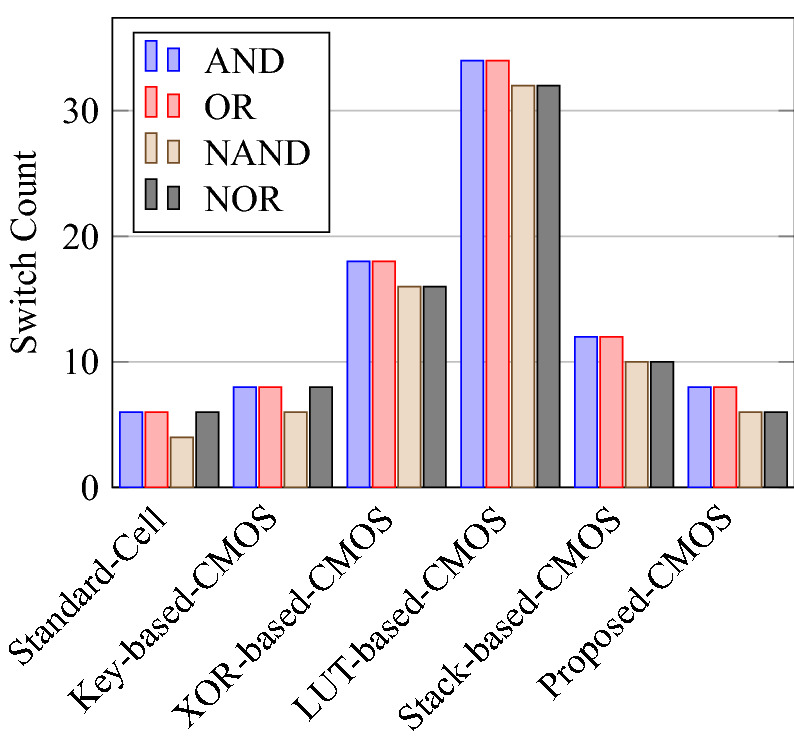
Switch count for gates.

**Figure 30:**
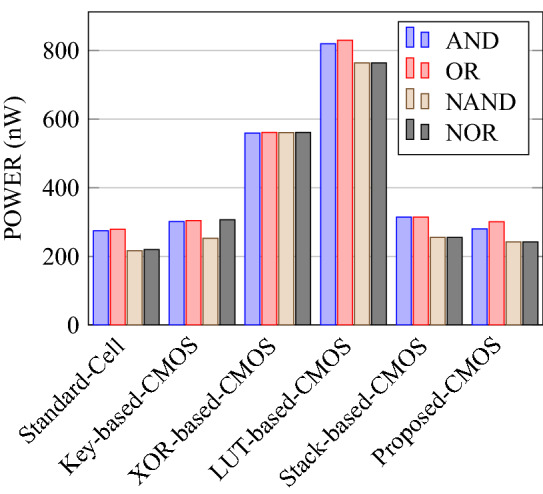
Total power consumed by each gate.

**Figure 31:**
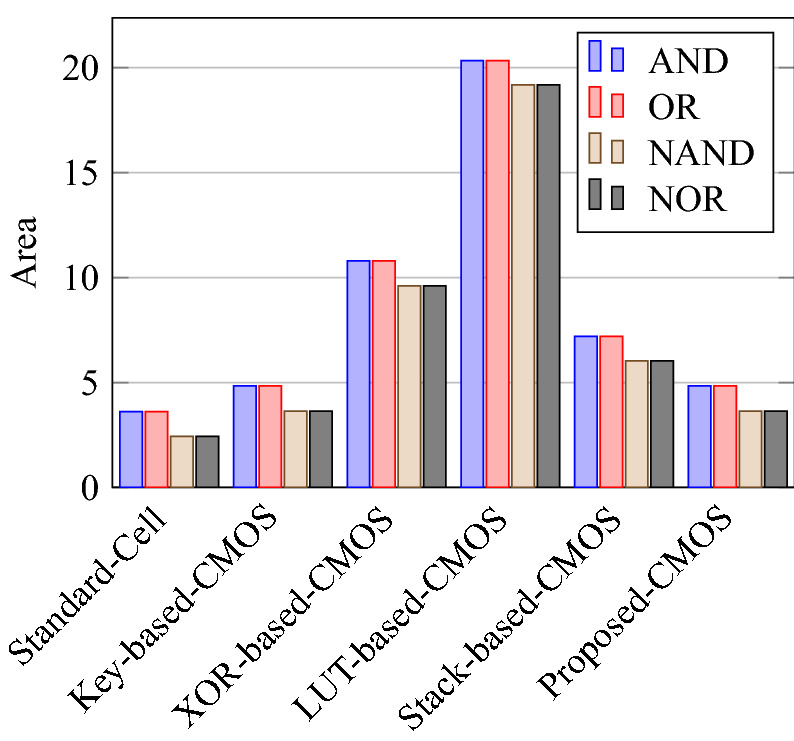
Total area consumed by each gate.

**Figure 32:**
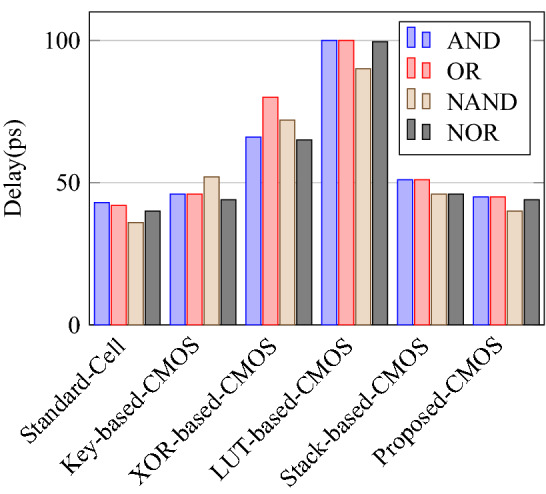
Delay of the each gate.

**Figure 33:**
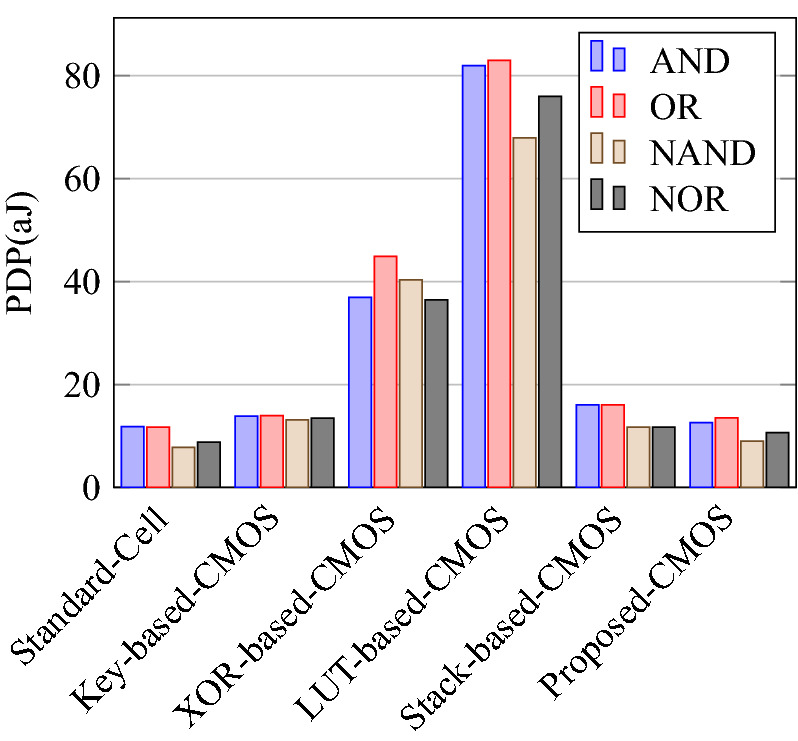
Power delay product of each gate.

**Figure 34:**
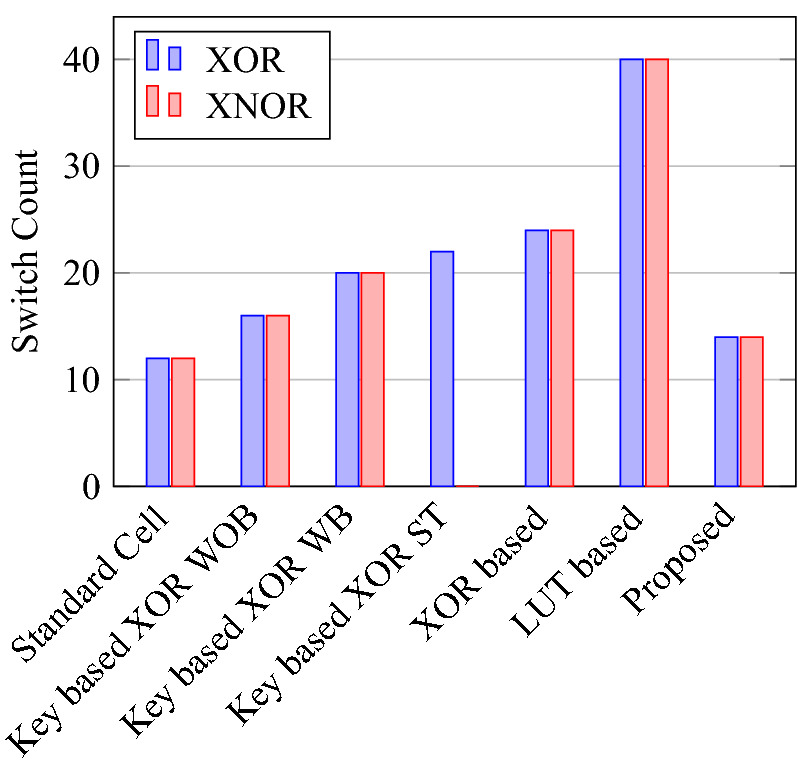
Switch count for XOR and XNOR.

**Figure 35:**
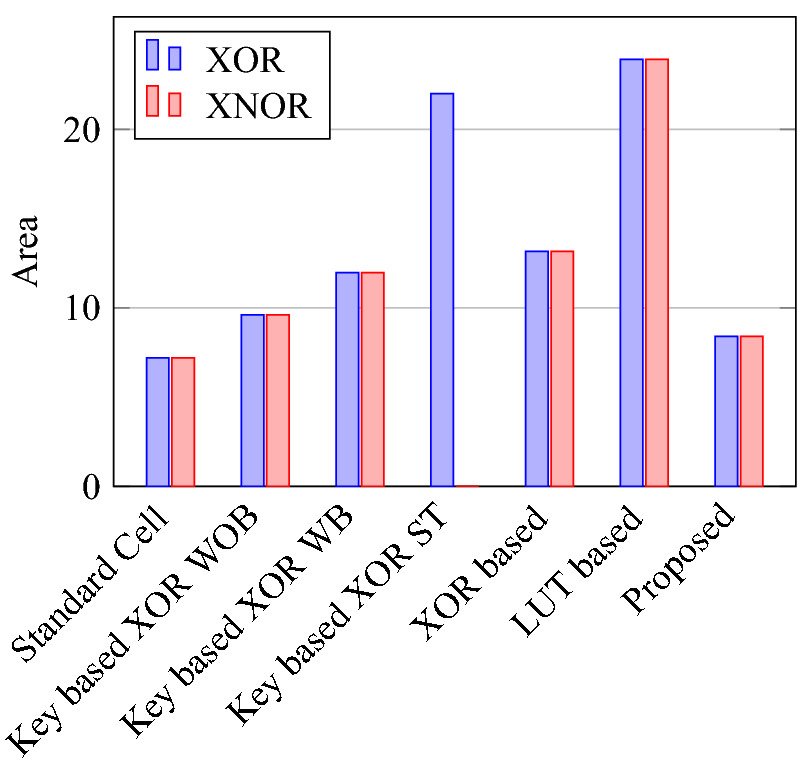
Area for XOR and XNOR gates.

**Figure 36:**
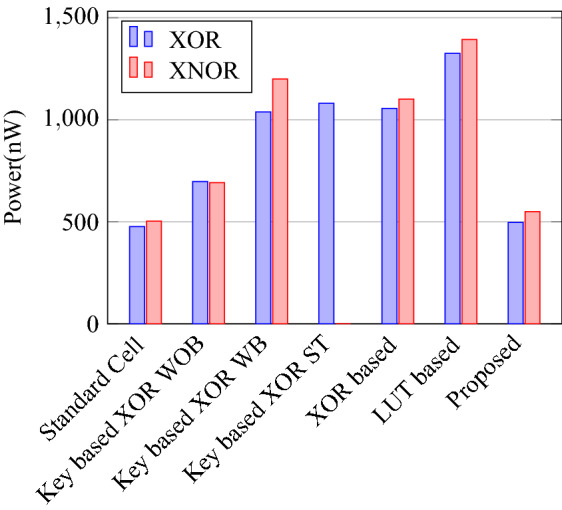
Power for XOR and XNOR gates.

**Figure 37:**
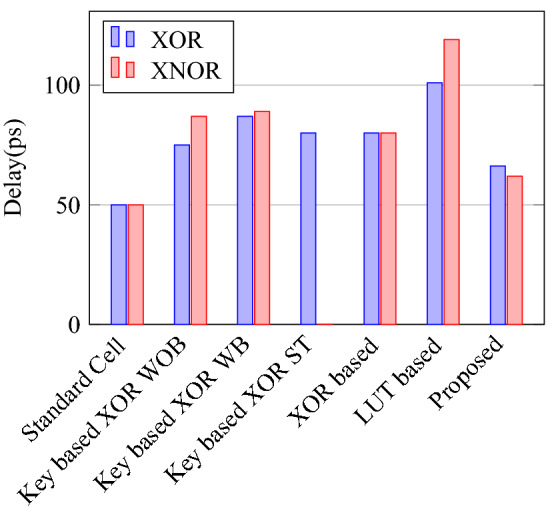
Delay for XOR ans XNOR gates.

**Figure 38:**
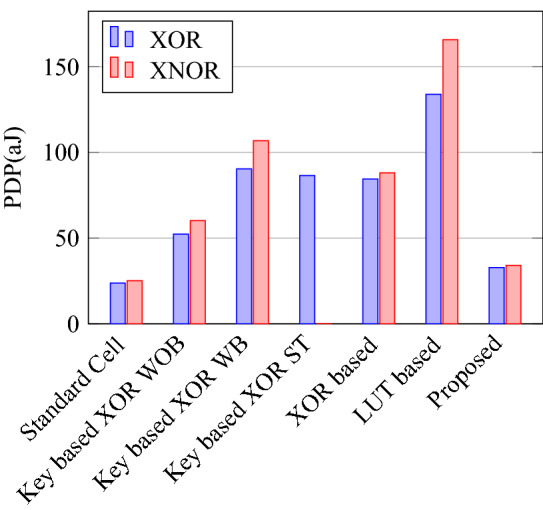
PDP for XOR ans XNOR gates.

On a similar basis, this analysis can be extended to the rest of the gates (OR, NAND, NOR, XOR, and XNOR), which are provided in the tabulations 18, 19, 20, 21, 22 and its corresponding graphs are shown in Figs. 29, 30, 31, 32, 33, 34, 35, 36, 37, 38 respectively. There is a certain percentage overhead observed when using corresponding literature circuits over the proposed circuits. This analysis proves the proposed gates’ efficiency in circuit fabrication.

**Table 11:** Results comparison for AND gate.

Encryption methodology	Switch count	Area, μm^2^	Power, nW	Delay, ps	PDP, aJ
Standard cell	6	3.62	274.6	43	11.80
Key based CMOS	8	4.84	301.5	46	13.86
XOR based CMOS	18	10.79	559.2	66	36.90
LUT based CMOS	34	20.34	819.5	100	81.95
Stack based CMOS	12	7.20	314.4	51	16.03
Proposed CMOS	8	4.84	280	45	12.6

**Table 12:** Results cmparison for OR gate.

Encryption methodology	Switch count	Area, μm^2^	Power, nW	Delay, ps	PDP, aJ
Standard cell	6	3.62	278.8	42	11.70
Key based CMOS	8	4.84	304.1	46	13.98
XOR based CMOS	18	10.79	560.9	80	44.87
LUT based CMOS	34	20.34	829.3	100	82.93
Stack based CMOS	12	7.20	314.4	51	16.03
Proposed CMOS	8	4.84	301	45	13.54

**Table 13:** Results comparison for NAND gate.

Encryption methodology	Switch count	Area, μm^2^	Power, nW	Delay, ps	PDP, aJ
Standard cell	4	2.44	216.5	36	7.79
Key based CMOS	6	3.64	252.6	52	13.13
XOR based CMOS	16	9.61	560.4	72	40.34
LUT based CMOS	32	19.18	754.4	90	67.89
Stack based CMOS	10	6.027	255.3	46	11.74
Proposed CMOS	6	3.64	225.7	40	9.02

**Table 14:** Results comparison for NOR gate.

Encryption methodology	Switch count	Area, μm^2^	Power, nW	Delay, ps	PDP, aJ
Standard cell	4	2.44	219.8	40	8.79
Key based CMOS	6	3.64	306.4	44	13.48
XOR based CMOS	16	9.61	560.8	65	36.45
LUT based CMOS	32	19.18	763.6	99.5	75.97
Stack based CMOS	10	6.027	255.3	46	11.74
Proposed CMOS	6	3.64	242	44	10.64

**Table 15:** Results comparison for XOR gate.

Encryption methodology	Switch count	Area, μm^2^	Power, nW	Delay, ps	PDP, aJ
Standard cell	12	7.20	476.8	50	23.84
Key based XOR PT without buffer	16	9.61	697.5	75	52.31
Key based XOR PT with buffer	20	11.97	1039	87	90.39
Key based XOR ST	22	13.17	1081	80	86.48
XOR based	24	14.36	1056	80	84.48
LUT based	40	23.91	1326	101	133.92
Stack based	–	–	–	–	–
Proposed XOR/OR	14	8.41	496.7	66.2	32.88

**Table 16:** Results comparison for XNOR gate.

Encryption methodology	Switch count	Area, μm^2^	Power, nW	Delay, ps	PDP, aJ
Standard cell	12	7.20	503.5	50	25.17
Key based XNOR PT without buffer	16	9.61	692.3	87	60.23
Key based XNOR PT with buffer	20	11.97	1200	89	106.8
XOR based	24	14.36	1101	80	88.08
LUT based	40	23.91	1393	119	165.76
Stack based	–	–	–	–	–
Proposed XNOR/AND	14	8.41	550	62	34.1

**Figure 39:**
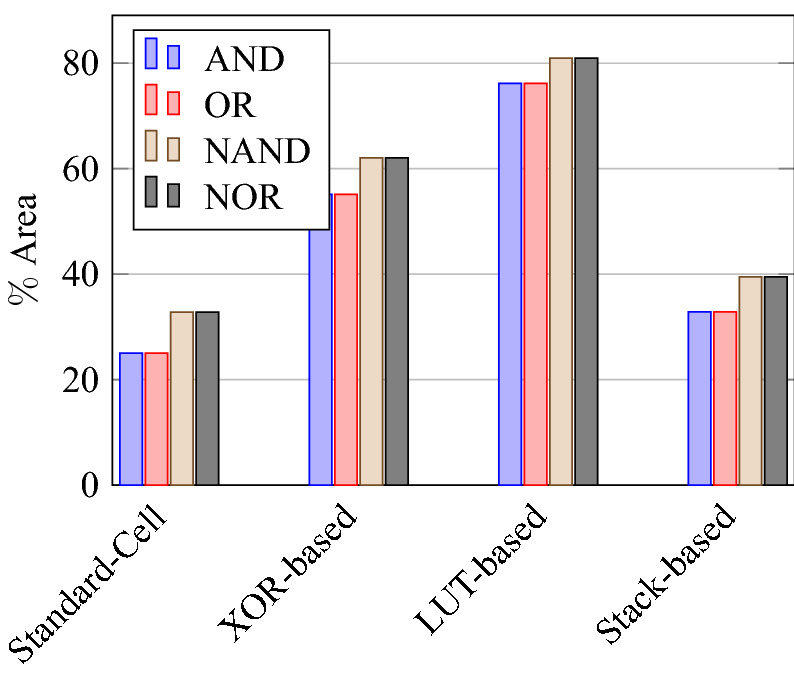
% Change in area compared to proposed design.

**Figure 40:**
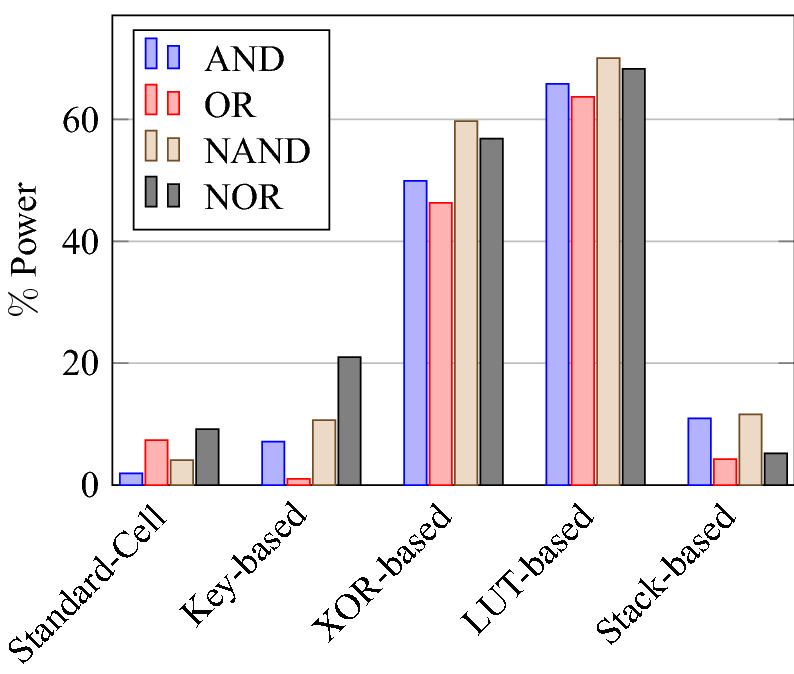
% Change in power compared to proposed design.

**Figure 41:**
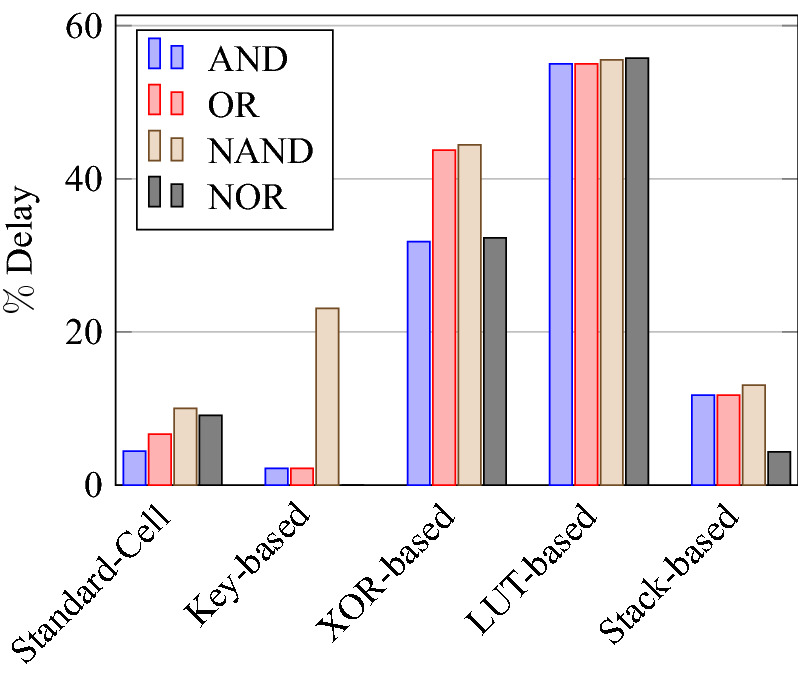
% Change in delay compared to proposed design.

**Figure 42:**
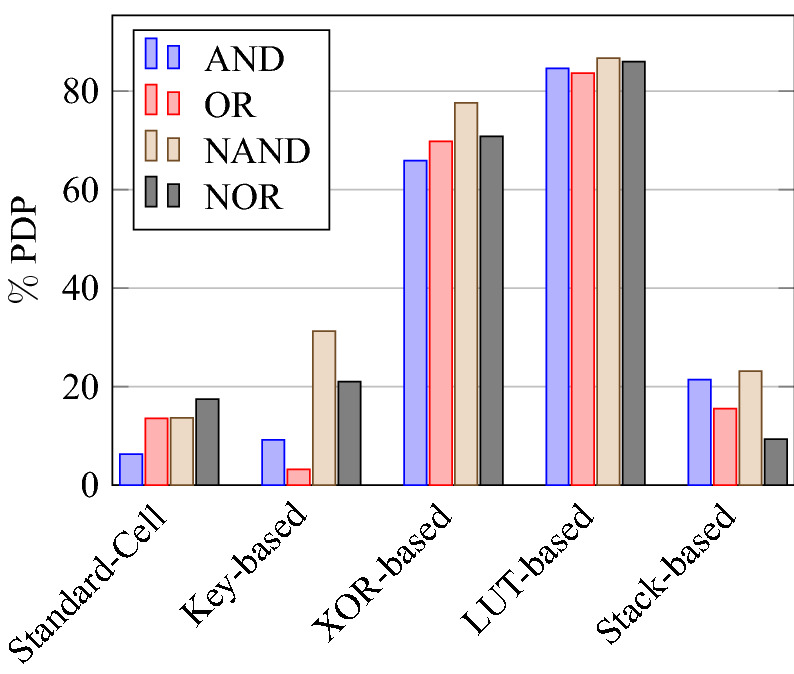
% Change in PDP compared to proposed design.

### Analysis of design overheads

This section presents the analysis of design overheads for each gate in a specific encryption topology. These overheads are percentage changes in the area, power, delay/performance, and energy. The corresponding gate parameters for the area, power, delay, and PDP are taken from the tabulations 11, 12, 13, 14, 15, 16. Corresponding percentage changes for each gate are taken from tabulations 17, 18, 19, 20, 21, 22. The percentage analysis for literature encryption methodologies are shown in the Tables from 23, 24, 25, 26 and its corresponding graphs are shown in Figs. 39, 40, 41, 42 respectively.

When considering XOR-based encryption analysis from Table 23, 52.87% reduction in area, 25.63% power saving, 32% performance improvement, and 67.73% overall energy saving on an average of all gates is observed when replacing XOR-based encrypted gates with proposed secure gates. Similarly, consider LUT-based logic encryption analysis from Table 24. There is almost a 74% reduction in area overhead, 65.16% power saving, 50.61% performance improvement, and 84.28% energy saving observed when replacing LUT-based gates with proposed secure gates.

**Figure 43:**
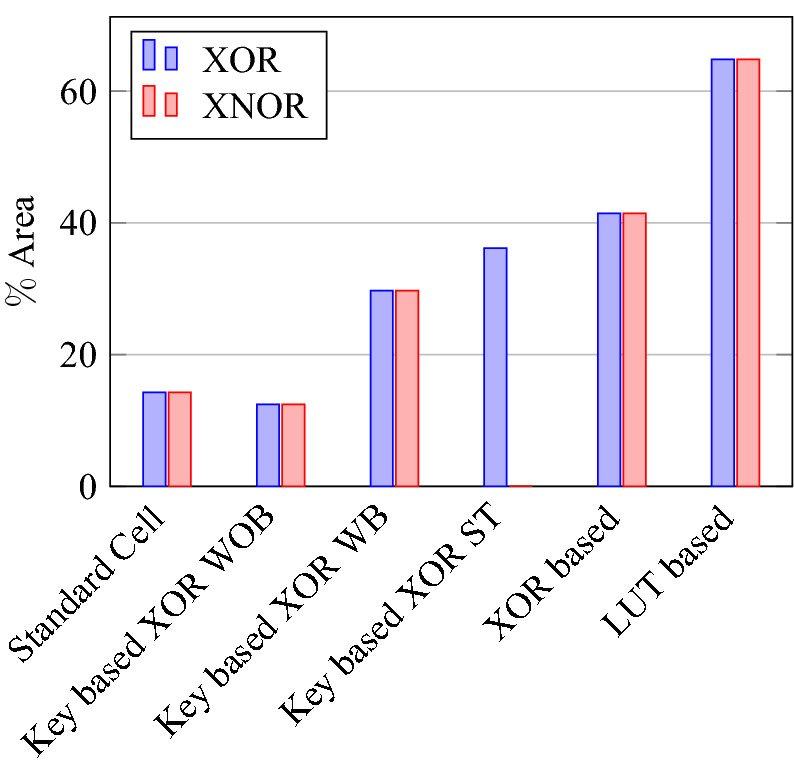
% of Change in area for XOR & XNOR gates compared to proposed design.

**Figure 44:**
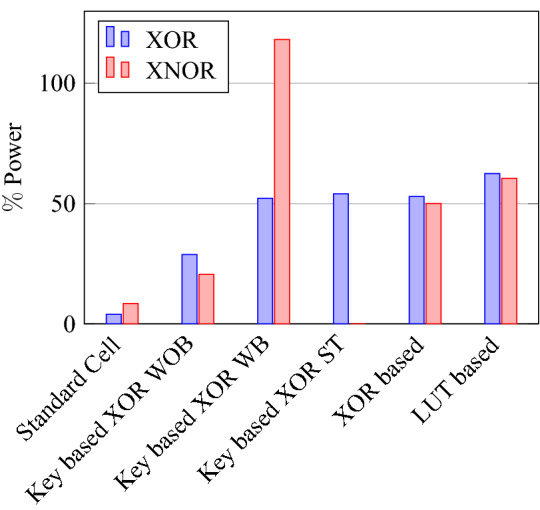
% of Change in power for XOR & XNOR gates compared to proposed design.

**Figure 45:**
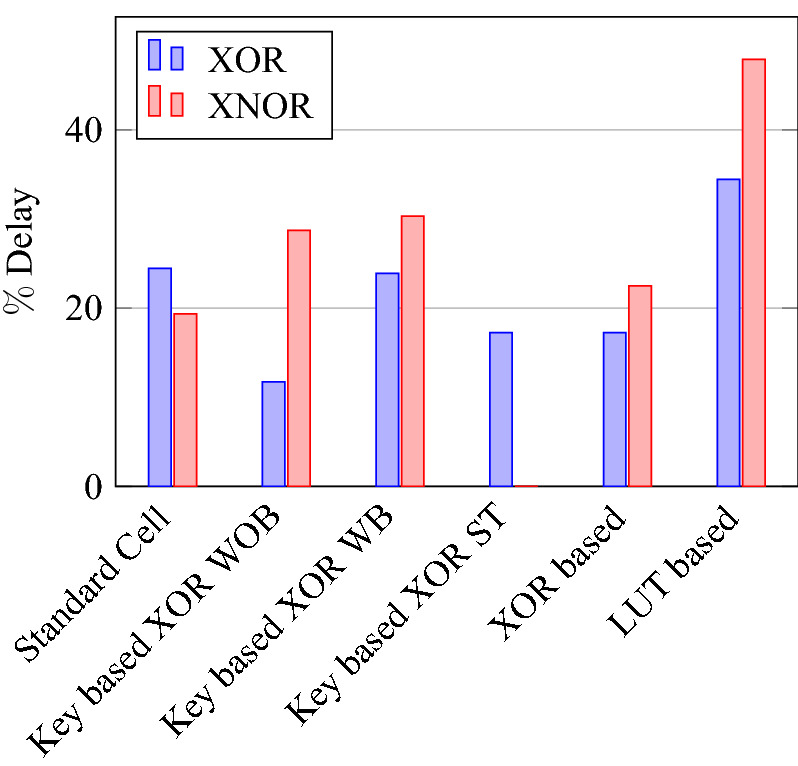
% of Change in delay for XOR & XNOR gates compared to proposed design.

**Figure 46:**
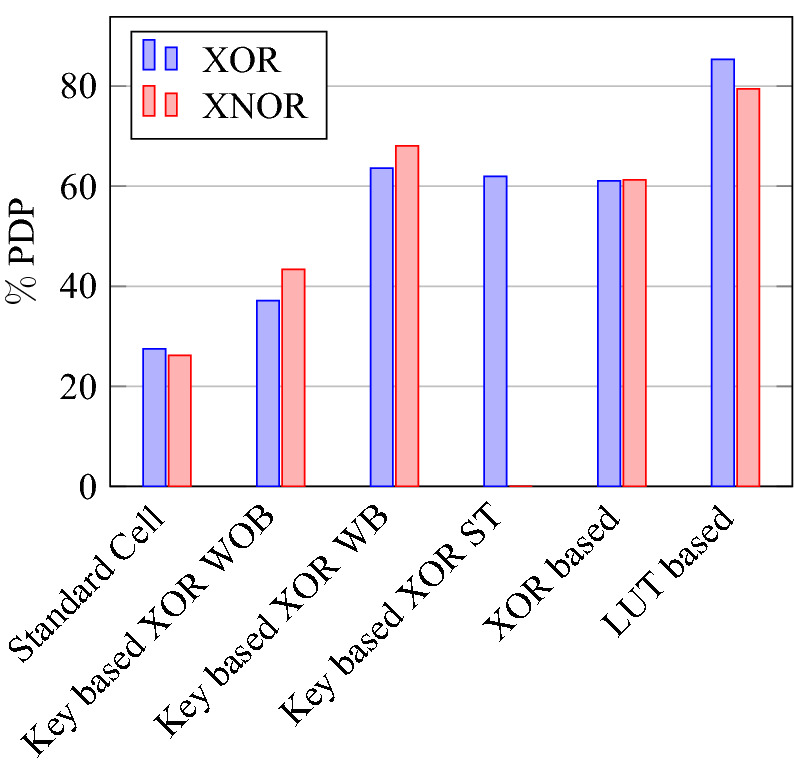
% of Change in PDP for XOR & XNOR gates compared to proposed design.

**Figure 47:**
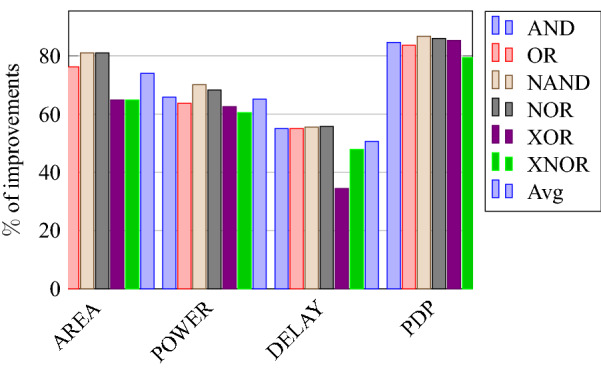
% of LUT overhead over the proposed encryption.

**Figure 48:**
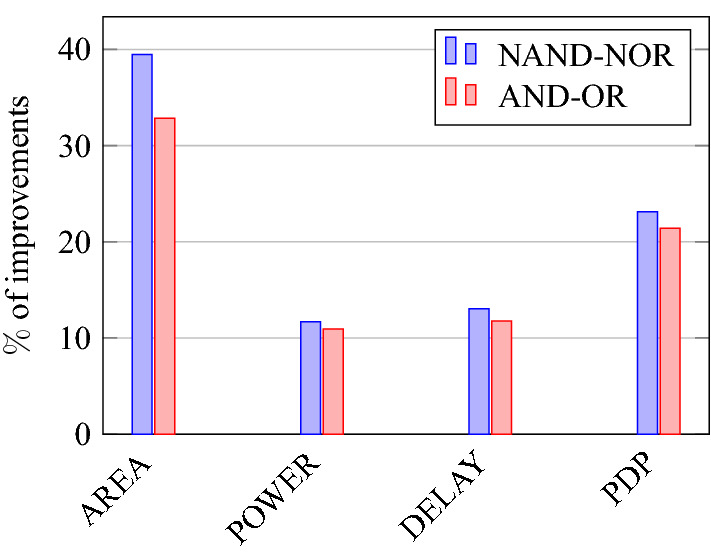
% of Stackbased overhead over the proposed encryption.

**Figure 49:**
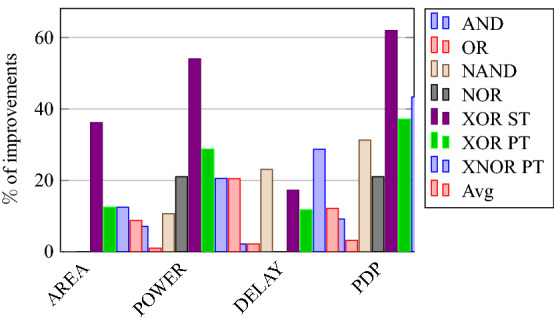
% of Overhead of keybased logic encrption over the proposed encryption.

**Figure 50:**
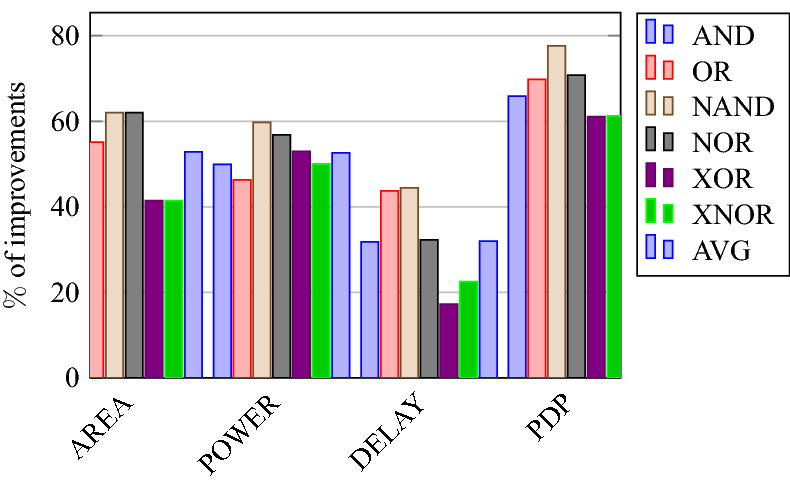
% of Overhead for XOR based over the proposed encryption.

On a similar basis, corresponding percentage improvements are observed when replacing stack-based and key-based literature circuit topologies with proposed gate topologies. Those results can be observed from tabulations 25 and 26. Since there are limited gates for stack-based topology, percentage analysis for those gates is stated in Table 25. The percentage of improvement in each parameter compared with the proposed designs are shown in the graphs from Figs. 43, 44, 45, 46, 47, 48, 49, 50 respectively.

As per the analysis, gate-level encrypted topologies are XOR-based and LUT-based encryption methods. Transistor-level encrypted topologies are stack-based and key-based methods. The efficiency of the proposed gates by considering the average of both the gate level methodologies, there is a 63.43% reduction in area overhead, 58.89% power saving, 41.30% performance improvement, and 76% energy saving observed. When considering the proposed gates over the average of both the transistor level methodologies, there is a 22.44% reduction in area, 15.85% power saving, 12.28% performance improvement, and 25.92% overall energy saving is observed as per the experimental analysis. Considering all literature circuit topologies (XOR, LUT, stack, and key-based), there is a 42.94% area reduction, 37.37% power savings, 26.79% performance upgrade, and 50.96% overall energy savings observed on an average. These overheads and their percentage analysis prove the proposed circuits’ efficacy in terms of adaptability, fabrication, reliability, and compatibility.

**Table 17:** % of Changes for AND gate.

Encryption methodology	% Area	I/R	% Power	I/R	% Delay	I/R	% PDP	I/R
Standard cell	25	R	1.92	R	4.44	R	6.287	R
Key based	0	–	7.13	I	2.173	I	9.149	I
XOR based	55.14	I	49.92	I	31.81	I	65.860	I
LUT based	76.20	I	65.83	I	55	I	84.624	I
Stack based	32.86	I	10.94	I	11.76	I	21.418	I

**Table 18:** % of Changes for OR gate.

Encryption methodology	% Area	I/R	% Power	I/R	% Delay	I/R	% PDP	I/R
Standard cell	25	R	7.37	R	6.66	R	13.55	R
Key based	0	–	1.01	I	2.17	I	3.17	I
XOR based	55.14	I	46.33	I	43.75	I	69.81	I
LUT based	76.20	I	63.70	I	55	I	83.66	I
Stack based	32.86	I	4.26	I	11.76	I	15.52	I

**Table 19:** % of Changes for NAND gate.

Encryption methodology	% Area	I/R	% Power	I/R	% Delay	I/R	% PDP	I/R
Standard cell	32.8	R	4.076	R	10	R	13.66	R
Key based	0	–	10.64	I	23.07	I	31.26	I
XOR based	62.03	I	59.72	I	44.44	I	77.62	I
LUT based	80.98	I	70.08	I	55.55	I	86.70	I
Stack based	39.47	I	11.59	I	13.04	I	23.12	I

**Table 20:** % of Changes for NOR gate.

Encryption methodology	% Area	I/R	% Power	I/R	% Delay	I/R	% PDP	I/R
Standard cell	32.8	R	9.17	R	9.09	R	17.43	R
Key based	0	–	21.01	I	0	–	21.01	I
XOR based	62.03	I	56.84	I	32.30	I	70.78	I
LUT based	80.98	I	68.30	I	55.77	I	85.98	I
Stack based	39.47	I	5.20	I	4.34	I	9.33	I

**Table 21:** % of Changes for XOR gate.

Encryption methodology	% Area	I/R	% Power	I/R	% Delay	I/R	% PDP	I/R
Standard cell	14.28	R	4.00	R	24.47	R	27.49	R
Key based XOR PT without Buffer	12.48	I	28.78	I	11.73	I	37.14	I
Key based XOR PT with Buffer	29.75	I	52.19	I	23.90	I	63.62	I
Key based XOR ST	36.17	I	54.05	I	17.25	I	61.97	I
XOR based	41.45	I	52.96	I	17.25	I	61.07	I
LUT based	64.82	I	62.54	I	34.45	I	85.31	I

**Table 22:** % of Changes for XNOR gate.

Encryption methodology	% Area	I/R	% Power	I/R	% Delay	I/R	% PDP	I/R
Standard cell	14.28	R	8.45	R	19.35	R	26.17	R
Key based XNOR PT without buffer	12.48	I	20.55	I	28.73	I	43.38	I
Key based XNOR PT with buffer	29.75	I	118.18	I	30.33	I	68.07	I
XOR based	41.45	I	50.04	I	22.5	I	61.28	I
LUT based	64.82	I	60.51	I	47.89	I	79.42	I

**Table 23:** Analysis of design overheads for XOR based logic encryption along with % of improvements when compared with proposed logic encryption.

Gate	Area (μm^2^)	Power (nW)	Delay (ps)	PDP (aJ)
AND	10.79 (55.14%)	559.2 (49.92%)	66 (31.81%)	36.90 (65.86%)
OR	10.79 (55.14%)	560.9 (46.33%)	80 (43.75%)	44.87 (69.81%)
NAND	9.61 (62.03%)	560.4 (59.72%)	72 (44.44%)	40.34 (77.62%)
NOR	9.61 (62.03%)	560.8 (56.84%)	65 (32.30%)	36.45 (70.78%)
XOR	14.36 (41.45%)	1056 (52.96%)	80 (17.25%)	84.48 (61.07%)
XNOR	14.36 (41.45%)	1101 (50.04%)	80 (22.5%)	88.08 (61.28%)
Average	52.87%	52.63%	32%	67.73%

**Table 24:** Analysis of design overheads for LUT based logic encryption along with % of improvements when compared with proposed logic encryption.

Gate	Area (μm^2^)	Power (nW)	Delay (ps)	PDP (aJ)
AND	20.34 (76.2%)	819.5 (65.83%)	100 (55%)	81.95 (84.62%)
OR	20.34 (76.2%)	829.3 (63.7%)	100 (55%)	82.93 (83.66%)
NAND	19.18 (80.98%)	754.4 (70.08%)	90 (55.55%)	67.89 (86.70%)
NOR	19.18 (80.98%)	763.6 (68.3%)	99.5 (55.77%)	75.97 (85.98%)
XOR	23.91 (64.82%)	1326 (62.54%)	101 (34.45%)	133.92 (85.31%)
XNOR	23.91 (64.82%)	1393 (60.51%)	119 (47.89%)	165.76 (79.42%)
Average	74%	65.16%	50.61%	84.28%

**Table 25:** Analysis of design overheads for Stack based logic encryption along with % of improvements when compared with proposed logic encryption.

Gate	Area (μm^2^)	Power (nW)	Delay (ps)	PDP (aJ)
NAND-NOR	6.027(39.47%)	255.3 (11.69%)	46 (13.04%)	11.74 (23.12%)
AND-OR	7.2 (32.86%)	314.4 (10.94%)	51 (11.76%)	16.03 (21.41%)
Average	36.16%	11.26%	12.4%	22.26%

**Table 26:** Analysis of design overheads for Key based logic encryption along with % of improvements when compared with proposed logic encryption.

Gate	Area (μm^2^)	Power (nW)	Delay (ps)	PDP (aJ)
AND	4.84 (0%)	301.5 (7.13%)	46 (2.173%)	13.86 (9.149%)
OR	4.84 (0%)	304.1 (1.01%)	46 (2.17%)	13.98 (3.17%)
NAND	3.64 (0%)	252.6 (10.64%)	52 (23.07%)	13.13 (31.26%)
NOR	3.64 (0%)	306.4 (21.01%)	44 (0%)	13.48 (21.01%)
XOR ST	13.17 (36.17%)	1081 (54.05%)	80 (17.25%)	86.48 (61.97%)
XOR PT	9.61 (12.48%)	697.5 (28.78%)	75 (11.73%)	52.31 (37.14%)
XNOR PT	9.61 (12.48%)	692.3 (20.55%)	87 (28.73%)	60.23 (43.38%)
Average	8.73%	20.45%	12.16%	29.58%

## Conclusion

In this paper, the need for hardware security and its crucial role in IC design is explained, stating the importance of hardware security by proposing a new topology for CMOS gates at the transistor level. The proposed topology is an efficient architecture for preventing IC piracy, overproduction, and reverse engineering. A detailed comparative study between the existing logic encryption methodologies and the proposed methodology is also explained. The results show an improvement of 42.94% reduction in area, 37.37% power saving, 26.79% performance improvement, and 50.96% energy savings on average compared with the existing design topologies. The proposed methodology provides a good trade-off between these design metrics alongside taking care of security features.

Researchers propose new algorithms and methodologies for hardware security in terms of logic encryption or logic locking. At the same time attacker also develops his ways of decrypting the encrypted circuits. Therefore, the security for an IC should be so that, whatever may be the path taken by an attacker, encrypted circuits have to provide a maximum amount of security against IC piracy, reverse engineering, and malicious tampering. To combat these hardware security issues, researchers, authors, and industry experts have to focus on methodologies like hardware hardening techniques, attacker strategies against encryption, and ensuring proper security at various levels of abstraction in circuits. Hence, we firmly believe that current work relies on trade-offs between security and circuit performance, and no defensive technique provides a 100% guarantee.

## Data Availability

The datasets generated during and/or analysed during the current study are available from the corresponding author on reasonable request.
